# Correction: Heterogeneity, Mixing, and the Spatial Scales of Mosquito-Borne Pathogen Transmission

**DOI:** 10.1371/journal.pcbi.1003540

**Published:** 2014-02-28

**Authors:** The PLOS Computational Biology Staff

## Notice of Republication

This article was republished on February 10, 2014, to replace an incorrectly published version. Please download this article again to view the correct version. The originally published, uncorrected article and the republished, corrected article are provided here for reference.

## Supporting Information

File S1 Originally published, uncorrected article
(PDF)

File S2 Republished corrected article
(PDF)
