# Supplementary material for: Heterogeneity, Mixing, and the Spatial Scales of Mosquito-Borne Pathogen Transmission
Source: PLoS Comput Biol. 2013 Dec 12;9(12):e1003327. doi: 10.1371/journal.pcbi.1003327 (PMC3861021; doi:10.1371/journal.pcbi.1003327)
Supplement: Text S1 — Additional details of the model and analysis. (PDF) [file pcbi.1003327.s001.pdf]

Supporting Information

for

Heterogeneity, Mixing, and the Spatial Scales of Mosquito-Borne Pathogen Transmission

T. Alex Perkins[1,2,*], Thomas W. Scott[1,2], Arnaud Le Menach[3], David L. Smith[1,4]

1 Fogarty International Center, National Institutes of Health, Bethesda, MD, USA

2 Department of Entomology, University of California, Davis, CA, USA

3 Center for Disease Dynamics, Economics and Policy, Washington, DC, USA

4 Department of Epidemiology, Johns Hopkins Bloomberg School of Public Health, Baltimore, MD, USA

∗ **E-mail: taperkins@ucdavis.edu**

Supporting Text S1. Additional details of model and analysis.

Mosquito population dynamics

The matrices L and F provide a partial description of mosquito population dynamics by accounting for adult mosquito movement and mortality. To complete the model of mosquito population dynamics, a model of the aquatic phase of the mosquito life cycle must be specified. The details of such a model will vary depending on the species in question, but two general features should be present in any such model: 1) recruitment at particular aquatic habitats is a function of how many adult females lay eggs there, and 2) survival during the aquatic phase is density dependent.

In addition to these basic requirements, we account for the fact that multiple feeding cycles elapse over the duration of the aquatic phase by specifying ξ aquatic stages. The aquatic phase begins with $A_{0,i}(t)$ eggs at larval habitat i and time t drawn from a Poisson distribution with mean equal to the number $M_i(t)$ of egg laying females multiplied by the average number v of eggs each female lays per feeding cycle. Over the course of each subsequent feeding cycle, the cohort progresses according to

$$A_{x+1,i}(t+1) = \text{Binomial}\left(A_{x,i}(t), (A_{x,i}(t)+1)^{\alpha_i - 1}\right), \tag{S1}$$

where α_i is a parameter that ranges between 0 and 1 and determines the strength of density dependence at larval habitat i. After ξ feeding cycles, $A_{\xi,i}(t+1)$ newly emerged adult females from larval habitat i go on to feed, lay eggs, move, and die according to F and L.

Under the assumption that adult females lay eggs exclusively at their natal larval habitats, an equilibrium can be found for the total number of adult females. If a proportion p survives each feeding cycle, then this equilibrium is

$$\overline{M} \approx \sum_{i \in \{f\}} \left(\frac{v^{\alpha_i^{\xi}}}{1-p} \right)^{\alpha_i^{\xi} - 1}. \tag{S2}$$

Relaxing the assumption that females lay eggs exclusively at their natal larval habitats invali-

dates this formula and complicates mosquito population dynamics considerably, but it nonetheless serves as a useful benchmark for interpreting the numerical significance of the parameters governing mosquito population dynamics.

Model implementation and parameterization

Values of some parameters used in numerical illustrations of the model were identical in all our analyses. These parameters are defined in Table S2. Code will be made available after publication upon request. In addition to common values of some parameters, one detail common to all implementations of the model was the representation of space. All blood-feeding habitats were constrained to a disc of radius $\pi^{-1/2}$. Random blood-feeding habitats were arranged on the disc according to a Poisson point process with the `rpoispp` function of the `spatstat` package [1] in R. Overdispersed blood-feeding habitats were arranged on the disc according to a Simple Sequential Inhibition process with the `rSSI` function of the `spatstat` package. Clustered blood-feeding habitats were arranged on the disc according to a Matérn process with the `rMatClust` function of the `spatstat` package. In all these cases, each aquatic habitat was associated with a blood-feeding habitat and was placed at a location drawn from a bivariate Gaussian distribution (mean = blood-feeding habitat location, variances = 10^{-4}, covariances = 0).

Although not strictly necessary under our framework, individual hosts were associated with home blood-feeding habitats in all implementations of the model. After blood-feeding habitats were defined, each was assigned a random number of resident hosts equal to 2 plus a Poisson random variable with $\lambda = 3.5$. A consequence of this choice is that the total number of hosts in the population varied somewhat among implementations of the model.

Another detail common to all implementations of the model was mosquito population dynamics. In stochastic simulations, mosquito population dynamics at aquatic habitats were modeled according to the description earlier in this Supplemental Text. Calculation of transmission metrics, however, depended on average values, $\overline{\Lambda}$, for the number of new adult females arising from each aquatic habitat per feeding cycle. Estimating $\overline{\Lambda}$ is not analytically tractable, as these values

depend in complicated ways on the geometry of $\{l\}$ and $\{f\}$, how many females lay eggs at each larval habitat, and how those eggs translate to emerging adult females after density-dependent mortality is exerted through the period of larval development. We therefore estimated $\overline{\Lambda}$ by simulating mosquito population dynamics across all $\{l\}$ and $\{f\}$ for 400 feeding cycles and taking the average of the number of emerging adult females per feeding cycle over feeding cycles 200 to 400, which appeared to display fluctuating but stable behavior.

Values of other parameters varied depending on assumptions about mosquito movement, host movement, and the biting attractiveness of hosts are available upon request. Details of the implementation of these assumptions are described below.

Well-mixed mosquito movement

Under the assumption of well-mixed mosquito movement, each mosquito has an equal probability of biting any individual host at any given time. Satisfying this assumption within our spatially explicit framework requires, in part, that each mosquito have an equal probability of moving from one habitat to any other habitat. To this end, each entry of L was equal to the survival between blood feeding and egg laying multiplied by $1/|l|$. Likewise, each entry of F was equal to the survival between egg laying and blood feeding multiplied by $1/|f|$.

Poorly mixed mosquito movement

Realistic algorithms for mosquito movement will vary tremendously for different species and in different ecological contexts. To illustrate one of the simplest possibilities, we adopted an algorithm in which 1) survival during movement between all habitat pairs was equal and 2) mosquitoes were more likely to move to nearby habitats than to ones farther away. Specifically, each entry of L was equal to the survival between blood feeding and egg laying multiplied by a Gaussian function (mean $= 0$, standard deviation $= 0.02 \cdot$ diameter of the disc on which the habitats are distributed) that was evaluated at the Euclidean distance between a blood-feeding habitat and all aquatic habitats and then normalized. The entries of F were populated in a

similar manner.

Well-mixed host movement

Under the assumption of well-mixed host movement, each host allocates its time evenly among all blood-feeding habitats. To reflect this assumption, each entry of H was set to $1/|f|$.

Poorly mixed host movement

As is the case for mosquitoes, realistic algorithms for individual host movement will vary tremendously for different host species and in different ecological contexts. To illustrate one such possibility, we modeled individual host movement after a crude representation of movement patterns in humans. The key features that we envisioned for such an algorithm are that human hosts have a home, they spend a substantial proportion of time there, they routinely visit only a few other locations, and they are more likely to visit locations closer to home than ones farther away. The specifics of how we implemented these assumptions are as follows. First, each host spent 50 percent of its time at its home blood-feeding habitat. Second, the remaining 50 percent of its time was allocated among a number of other blood-feeding habitats specified by a Weibull random variable (shape = 2, scale = 1.5). Non-home blood-feeding habitats frequented by a given host were selected randomly with normalized probabilities proportional to the values of a Gaussian function (mean = 0, standard deviation = 0.05 $\cdot$ diameter of the disc on which the habitats are distributed) evaluated at the Euclidean distances of all non-home blood-feeding habitats from home. Hosts allocated time at each of these non-home locations proportional to the values of the same Gaussian function. Finally, the entries of H were populated accordingly.

Scaling up from individuals to patches

For some analyses or applications it may be desirable to use a model defined at a more macroscopic scale than the level of individual blood-feeding habitats. Let $\{f\}$ denote a set of blood-feeding-habitat groups, such that each blood-feeding habitat in $\{f\}$ belongs to one and only one

member of $\{\mathscr{f}\}$. This grouping of blood-feeding habitats could be accomplished by tessellating over space, clustering by spatial or social-network distance, or by some other means. Furthermore, assume that each host has a home blood-feeding habitat at which it spends more time than others. There is thus a map of $\{h\}$ onto $\{f\}$ and in turn onto $\{\mathscr{f}\}$. This results in a grouping of hosts, $\{\mathscr{h}\}$, corresponding to the grouping of blood-feeding habitats.

A model over $\{\mathscr{f}\}$ and $\{\mathscr{h}\}$ can be parameterized with block versions of the matrices describing movement, including L, F, and U. Block versions of the metrics we derived, including B, Q, P, V, and R, could also be specified on $\{\mathscr{f}\}$ and $\{\mathscr{h}\}$ in this way. On-diagonal blocks describe movement within a group, and off-diagonal blocks describe movement among groups. Each of the state-variable vectors specified over $\{f\}$ can be condensed by specifying vectors of length $|\mathscr{f}|$ in which the $\mathscr{i}^{\text{th}}$ element is the sum of the states in elements $i \in \mathscr{i}$ from the vectors specified on $\{f\}$. Likewise, instead of tracking the infection status of each host with binary vectors, the variables pertaining to host infection dynamics on $\{\mathscr{h}\}$ denote the numbers of hosts in each infection state whose homes belong to each blood-feeding-habitat group.

The dynamics of blood-feeding-habitat groups can be described by a deterministic patch or metapopulation model of the expected values of each state variable in each group $\mathscr{f}_{\mathscr{i}}$ and $\mathscr{h}_{\mathscr{k}}$. This model's parameters result from summing or averaging parameters of the individual-based model across members of each group:

$$\mathcal{L}_{\mathscr{i},\mathscr{j}} = \sum_{i: f_i \in \mathscr{f}_{\mathscr{i}}} \frac{L_{i,j}}{|\mathscr{f}_{\mathscr{i}}|} \tag{S3a}$$

$$\mathcal{F}_{\mathscr{i},\mathscr{j}} = \sum_{j: f_j \in \mathscr{f}_{\mathscr{j}}} F_{i,j} \tag{S3b}$$

$$\mathcal{U}_{\mathscr{i},\mathscr{j}} = \sum_{k: h_k \in \mathscr{h}_{\mathscr{k}}} \sum_{i: f_i \in \mathscr{f}_{\mathscr{i}}} \frac{U_{k,i}}{|\mathscr{f}_{\mathscr{i}}|}. \tag{S3c}$$

These matrices are then used to specify dynamics similar to eq. (3) for hosts,

$$\mathcal{S}(t+1) = \mathcal{S}(t)(1-b)^{\sum_i \mathcal{I}_i'(t)\mathcal{U}} \tag{S4a}$$

$$\mathcal{E}_0(t+1) = \mathcal{S}(t)\left(1-(1-b)^{\sum_i \mathcal{I}_i'(t)\mathcal{U}}\right) \tag{S4b}$$

$$\mathcal{E}_e(t+1) = \mathcal{E}_{e-1}(t) \tag{S4c}$$

$$\mathcal{I}_0(t+1) = \mathcal{E}_{\sigma-1}(t) \tag{S4d}$$

$$\mathcal{I}_i(t+1) = \mathcal{I}_{i-1}(t)(1-\rho_{\text{fail}}(i-1)) \tag{S4e}$$

$$\mathcal{R}(t+1) = \mathcal{R}(t) + \sum_i \mathcal{I}_{i-1}(t)\rho_{\text{fail}}(i-1), \tag{S4f}$$

and eq. (4) for mosquitoes,

$$\mathcal{S}'(t+1) = (1-c)\mathcal{I}(t)\mathcal{U}^T\left(\Lambda(t+1)\mathcal{F} + \mathcal{S}'(t)\mathcal{L}\mathcal{F}\right) \tag{S5a}$$

$$\mathcal{E}_0'(t+1) = c\mathcal{I}(t)\mathcal{U}^T\left(\Lambda(t+1)\mathcal{F} + \mathcal{S}'(t)\mathcal{L}\mathcal{F}\right) \tag{S5b}$$

$$\mathcal{E}_e'(t+1) = \mathcal{E}_{e-1}'(t)\mathcal{L}\mathcal{F} \tag{S5c}$$

$$\mathcal{I}'(t+1) = \left(\mathcal{I}'(t) + \mathcal{E}_{\tau-1}'(t)\right)\mathcal{L}\mathcal{F}, \tag{S5d}$$

where Λ is a vector on $\{f\}$ representing the number of newly emerged adult female mosquitoes feeding somewhere in each feeding-station group. A definition of $\Lambda(t)$ consistent with our model of mosquito population dynamics is

$$\Lambda(t) = \left(\mathcal{M}(t-\xi)\mathcal{L}v\right)^{\alpha^{\xi}}\mathcal{F}, \tag{S6}$$

where $\mathcal{M}$ is a vector on $\{f\}$ representing the abundance of adult female mosquitoes in each feeding station group.

References

1. Baddeley A, Turner R (2005) Spatstat: an R package for analyzing spatial point patterns. Journal of Statistical Software 12: 1–42.

Table S1. Parameters and metrics defined as vectors or matrices.

Symbol	Definition	Equation	Dimensions
γ	Host biting suitability	–	$\|h\|$
Λ	Expected number of new adult female mosquitoes arising from each aquatic habitat per feeding cycle	–	$\|l\|$
B	Expected number of blood meals per feeding cycle on each host at each blood-feeding habitat	6	$\|f\| \times \|h\|$
H	Proportional allocation of each host's time across blood-feeding habitats	–	$\|h\| \times \|f\|$
M	Expected number of bites per feeding cycle at each blood-feeding habitat	5	$\|f\|$
P	Probability distribution of where secondary bites occur on hosts that received a primary bite at a given blood-feeding habitat	11	$\|f\| \times \|f\|$
Q	Expected number of potentially infectious bites at each blood-feeding habitat that originated from a single mosquito infected at some other blood-feeding habitat	7	$\|f\| \times \|f\|$
R	Probability of a secondary infection in a host arising from a primary infection in some other host	14	$\|h\| \times \|h\|$
r	Expected number of secondary host infections arising from a primary infection in a given host	15	$\|h\|$
U	Proportional distribution of bites at each blood-feeding habitat across hosts	2	$\|f\| \times \|h\|$
V	Expected number of secondary bites on a host arising from primary bites on some other host in a single feeding cycle	12	$\|h\| \times \|h\|$
v	Expected number of secondary bites on all hosts arising from primary bites on a given host in a single feeding cycle	13	$\|h\|$

Table S2. Parameters defined as scalar quantities.

Symbol	Definition
α	Strength of density dependence on larval mosquitoes
ν	Number of mosquito eggs per capita per feeding cycle
ξ	Length of the larval stage (in feeding cycles)
ρ	Duration of host infectiousness
σ	Pathogen incubation period in hosts
τ	Pathogen incubation period in mosquitoes
ψ	Proportion of blood meals taken on the focal host species
b	Mosquito-to-host transmission efficiency
c	Host-to-mosquito transmission efficiency
$\|f\|$	Number of blood-feeding habitats
$\|h\|$	Number of hosts
$\|l\|$	Number of aquatic habitats
s_F	Mosquito survival between egg laying and blood feeding
s_L	Mosquito survival between blood feeding and egg laying
