# Supplementary material for: Correction: Heterogeneity, Mixing, and the Spatial Scales of Mosquito-Borne Pathogen Transmission
Source: PLoS Comput Biol. 2014 Feb 28;10(2):e1003540. doi: 10.1371/journal.pcbi.1003540 (PMC3938404; doi:10.1371/journal.pcbi.1003540)
Supplement: File S1 — Originally published, uncorrected article (PDF) [file pcbi.1003540.s001.pdf]

OPEN ACCESS Freely available online
PLOS COMPUTATIONAL BIOLOGY

Heterogeneity, Mixing, and the Spatial Scales of Mosquito-Borne Pathogen Transmission

T. Alex Perkins[1,2]*, Thomas W. Scott[1,2], Arnaud Le Menach[3], David L. Smith[1,3,4]

1 Fogarty International Center, National Institutes of Health, Bethesda, Maryland, United States of America, 2 Department of Entomology, University of California, Davis, California, United States of America, 3 Center for Disease Dynamics, Economics and Policy, Washington, D.C., United States of America, 4 Department of Epidemiology, Johns Hopkins Bloomberg School of Public Health, Baltimore, Maryland, United States of America

Abstract

The Ross-Macdonald model has dominated theory for mosquito-borne pathogen transmission dynamics and control for over a century. The model, like many other basic population models, makes the mathematically convenient assumption that populations are well mixed; *i.e.*, that each mosquito is equally likely to bite any vertebrate host. This assumption raises questions about the validity and utility of current theory because it is in conflict with preponderant empirical evidence that transmission is heterogeneous. Here, we propose a new dynamic framework that is realistic enough to describe biological causes of heterogeneous transmission of mosquito-borne pathogens of humans, yet tractable enough to provide a basis for developing and improving general theory. The framework is based on the ecological context of mosquito blood meals and the fine-scale movements of individual mosquitoes and human hosts that give rise to heterogeneous transmission. Using this framework, we describe pathogen dispersion in terms of individual-level analogues of two classical quantities: vectorial capacity and the basic reproductive number, R_0. Importantly, this framework explicitly accounts for three key components of overall heterogeneity in transmission: heterogeneous exposure, poor mixing, and finite host numbers. Using these tools, we propose two ways of characterizing the spatial scales of transmission—pathogen dispersion kernels and the evenness of mixing across scales of aggregation—and demonstrate the consequences of a model's choice of spatial scale for epidemic dynamics and for estimation of R_0, both by a priori model formulas and by inference of the force of infection from time-series data.

Citation: Perkins TA, Scott TW, Le Menach A, Smith DL (2013) Heterogeneity, Mixing, and the Spatial Scales of Mosquito-Borne Pathogen Transmission. PLoS Comput Biol 9(12): e1003327. doi:10.1371/journal.pcbi.1003327

Editor: Marcel Salathé, Pennsylvania State University, United States of America

Received August 6, 2013; **Accepted** September 24, 2013; **Published** December 12, 2013

Copyright: © 2013 Perkins et al. This is an open-access article distributed under the terms of the Creative Commons Attribution License, which permits unrestricted use, distribution, and reproduction in any medium, provided the original author and source are credited.

Funding: This work was supported by the Research and Policy for Infectious Disease Dynamics (RAPIDD) program of the Science and Technology Directory, Department of Homeland Security, and Fogarty International Center, National Institutes of Health. DLS and ALM (49446) and TWS (OPP52250) acknowledge funding from the Bill and Melinda Gates Foundation. DLS (U19AI089674) and TWS (R01 AI069341) acknowledge funding from the NIH/NIAID. DLS acknowledges funding from the Bloomberg Family Foundation. The funders had no role in study design, data collection and analysis, decision to publish, or preparation of the manuscript.

Competing Interests: The authors have declared that no competing interests exist.

* E-mail: taperkins@ucdavis.edu

Introduction

Dynamic models of mosquito-borne pathogens are being used in scientific research to investigate the mechanisms and processes underlying transmission and in policy research to give advice about malaria elimination and global malaria eradication [1–4], stratification of a country to improve disease control [5–9], strategies for managing the evolution of resistance to insecticides and antimalarial drugs [10,11], and ideal properties, potential impact, and delivery strategies for new vaccines, drugs, and vector control technologies [5,12–14]. Giving robust policy advice on these issues requires extending the current evidence base and theory to weigh various sources of heterogeneity, which are known to affect transmission of mosquito-borne and other pathogens. The first mathematical models of directly transmitted infectious agents and mosquito-borne pathogens [15,16], as well as most subsequent models, have nonetheless assumed that transmission obeys the law of mass action [17], a convenient mathematical formulation that was first developed to model chemical reactions and has since been applied in a wide range of other contexts. Mass action assumes that encounters in a very large population are so well mixed that different types interact randomly and in direct proportion to their densities. Whereas this assumption may be suitable for modeling infectious diseases in some contexts, it is also important to know when the mass-action assumption breaks down. Here, we develop a new mathematical framework capable of assessing the appropriateness of the mass-action paradigm at different spatial scales and investigating the biological heterogeneities underpinning these scaling relationships.

Heterogeneous transmission of pathogens is a pervasive issue. In populations afflicted by sexually transmitted diseases, certain individuals engage in sexual activity more frequently and with different partners than others [18]. With respect to other types of directly transmitted diseases, individuals come into contact with limited subsets of their population depending on patterns of routine movement or social relationships [19,20]. Such individual variation in contact patterns means that some individuals play a much more important role in transmission than others, which has considerable implications for the emergence, spread, persistence, epidemiology, and control of pathogens [21–25].

Transmission of mosquito-borne pathogens is also heterogeneous. At relatively coarse scales, transmission heterogeneity has

Author Summary

Pathogens transmitted by mosquitoes, such as malaria and dengue, are notorious for the biological complexity associated with how they are transmitted within local communities. Yet mathematical models for these pathogens, which are critical tools for making recommendations for control policy, are based around concepts originally designed to describe how molecules interact in chemical systems. To provide those interested in mosquito-borne diseases a more appropriate tool for modeling their transmission, we introduce a mathematical framework that is based on the spatial locations where mosquitoes lay eggs and feed on blood and how mosquitoes and hosts move about those locations. Analysis of this framework shows that the transmission contributions of different hosts and locations can be calculated, and that overall potential for transmission in a community depends on three concepts: heterogeneous exposure (some people bitten by mosquitoes more than others), poor mixing (non-random contacts between hosts and mosquitoes), and finite population sizes (each host can contribute at most one new infection towards the population total). Together, these factors determine critical levels of vaccination coverage to eliminate a pathogen and the spatial areas over which transmission should be modeled and studied in the field.

been described in terms of spatial "hotspots" [26], whereas at finer scales it has been described in terms of heterogeneous biting: the highly skewed distribution of biting in which 20% of the host population gets 80% or more of all the bites [22]. At those fine scales, DNA profiling of mosquito blood meals provides direct evidence for heterogeneous biting by the mosquitoes that transmit filarial worms, dengue virus, and malaria parasites [27–29]. Studies have shown that heterogeneous mosquito biting is associated with human body size [30,31], defensive behavior [32,33], pregnancy [34,35], blood type [36], alcohol consumption [37,38], and some volatile chemicals [39] found in breath and sweat [40–43]. Other studies have found that heterogeneity exists among households due to factors such as proximity to the aquatic habitats of immature mosquitoes [26], the type of house [44,45], the prevailing direction of the wind [46], and other factors associated with mosquito movement patterns [46–48]. Yet others have proposed that the patterns of routine movement by hosts may put some at greater risk of exposure to mosquitoes than others [49,50]. Specifically, hosts that spend more time at locations with high mosquito densities at times when mosquitoes are actively biting have a greater risk of being bitten [51–53]. Altogether, abundant evidence from decades of empirical research shows that pathogen transmission is highly heterogeneous at a variety of scales, that it has many causes, and that it is epidemiologically important for mosquito-borne pathogens [24,25,54].

Despite the ubiquitous evidence for heterogeneous transmission, mathematical models of mosquito-borne pathogen transmission rarely consider these complexities and usually assume mass action [17]. There are, of course, a number of notable exceptions to this rule, including [24,25,54–57] and especially [21,58], which provide general theoretical insights about the impact of heterogeneous transmission on mosquito-borne pathogen transmission. Unfortunately though, the impact of this work on modeling and policy for mosquito-borne diseases has been limited [17], likely because of the lack of a clear path to connect these rather abstract models to the complex biology of real systems. Instead, much recent attention has focused on the development of simulation-based models [59–65], which incorporate a great deal of biological complexity but do so at the expense of the analytical tractability and broad insight afforded by [21,58] and others. An intermediary set of tools striking an appropriate balance between the transparency of simple, abstract models and the complexity of simulation-based models could provide a useful shortcut to basic insights on which policy decisions often depend.

In addition to their academic intrigue, these questions about heterogeneity and mixing are highly relevant for measurement of transmission and application of theory to real problems. One of the ways in which patterns of heterogeneity and mixing most directly influence the interface of theory and reality is by how they determine the spatial scales that characterize transmission. Without theory to inform practitioners about realistic scales of transmission, how are decisions to be made about the appropriate sampling frame for making valid inference about transmission? How are decisions to be made about basic questions such as the sample sizes required to achieve an appropriate degree of accuracy or precision for measurements to inform target intervention coverage levels or disease control measures? Characterizing the spatial scales of transmission is also relevant for designing randomized control trials for transmission blocking malaria vaccines and tetravalent dengue vaccines where study populations are influenced by surrounding populations, as well as for understanding the causes and consequences of the area effects observed in some bed net trials [66,67]. Theory and practice for mosquito-borne pathogen transmission and control thus requires a better characterization of heterogeneity, mixing, and the spatial scales that characterize transmission and control.

To address these gaps about heterogeneity, mixing, and the appropriate spatial scales for measuring and modeling transmission, we took a reductionist perspective and developed a mathematical framework that is based on the ecological context of encounters between adult female mosquitoes and their vertebrate hosts. From this, we derived mathematical formulas describing heterogeneous biting, transmission thresholds, and the spatial scales of transmission. In particular, we calculate next-generation matrices, individual reproductive numbers, and the population-level basic reproductive number, R_0. These approximations build on previous work on heterogeneous biting [21,58], but utilize a more mechanistic biological model and account for an inherent nonlinearity posed by finite host numbers, as in [68]. Leveraging the spatial specificity of our model, we demonstrate how these matrices can be used to characterize the spatial scales of transmission, and we provide examples of how models with inappropriate assumptions about these scales can lead to faulty prediction and inference.

Results

The Ross-Macdonald model assumes well-mixed transmission of a pathogen within a host population of indeterminate size. The potential intensity of transmission is determined by five parameters: (1) the population density of mosquitoes; (2) mosquito survival; (3) the time required to complete one cycle of mosquito feeding and egg laying; (4) the propensity for mosquitoes to feed on the pathogen's vertebrate host; and (5) the pathogen's extrinsic incubation period in the mosquito [5,69]. These five parameters comprise vectorial capacity, also known as the daily reproductive rate, which is defined as the expected number of infectious bites that could arise (assuming perfectly efficient pathogen transmission to mosquitoes from the vertebrate host) from all the mosquitoes that bite a single host on a single day. This parsimonious model has been widely used [17], but it is difficult to modify this simple

framework as a tool for investigating transmission because key aspects of mosquito behavior, including blood feeding and the corresponding host behaviors, are treated phenomenologically.

We developed a new mechanistic, mathematical framework for modeling the micro-epidemiology of mosquito-borne pathogen transmission. Our model is based on the biological activities of the mosquitoes and hosts—particularly their movements—that allow a pathogen to disperse, and it was formulated in a way that makes it possible to analyze transmission mathematically (Fig. 1). Our model is capable of utilizing very detailed information about transmission in a particular place, but the level of detail that could be used to fully calibrate models of this sort in a single place exceeds the capacity of field biologists to measure it. Rather than serving as a focal point for calibration, then, our purpose was to design a model that is flexible enough that it can serve as a tool for conducting experiments *in silico* to identify the biological details that are most relevant for transmission dynamics and disease control.

We also developed a parallel mathematical framework for modeling the macro-epidemiology of a mosquito-borne pathogen; *i.e.*, a patch-based or metapopulation model (*e.g.*, [70]). This macro-epidemiological model serves as a bridge between the complex, detailed micro-epidemiological framework and more recent patch-based Ross-Macdonald-like models, and we have developed utilities to map the models onto one another in limiting cases. The following sections describe the factors that give rise to heterogeneous biting, matrices describing the networks along which pathogens disperse, and concepts and metrics to measure the scales of transmission. Within this framework, we also simulate transmission and compare different criteria for the critical fraction of a population that must be vaccinated for a pathogen to be unable to sustain endemic transmission.

Heterogeneous Biting

The Ross-Macdonald model assumes homogeneous biting: a mosquito is equally likely to bite any individual in the vertebrate host population. Most evidence suggests that biting is highly heterogeneous, and that heterogeneous biting is an important quantitative feature of transmission. The framework we devised gives a mathematical description of three distinct processes that give rise to heterogeneous biting: the distribution of mosquito biting among different places, the number of hosts present at those places, and a rule describing how the bites are allocated among the hosts who are present. The way these assumptions manifest mathematically and their relevance to transmission dynamics are discussed in detail in the Methods section.

Mosquito blood meals are the focal event in pathogen transmission, and our model is based on a mathematical construct describing where and when mosquito-host encounters occur. Many mosquitoes have well-defined haunts and blood-feeding habits, such that blood feeding tends to occur in places that can be reasonably well characterized. These are rigorously described as a set of points, denoted $\{f\}$, containing $|f|$ objects, called blood-feeding habitats. A way of defining $\{f\}$ could be to consider the collection of homes in a city, which might be appropriate for human populations afflicted by malaria or dengue. Another, more flexible option would be to impose a lattice over an area and aggregate blood-feeding that takes place nearest to each point on

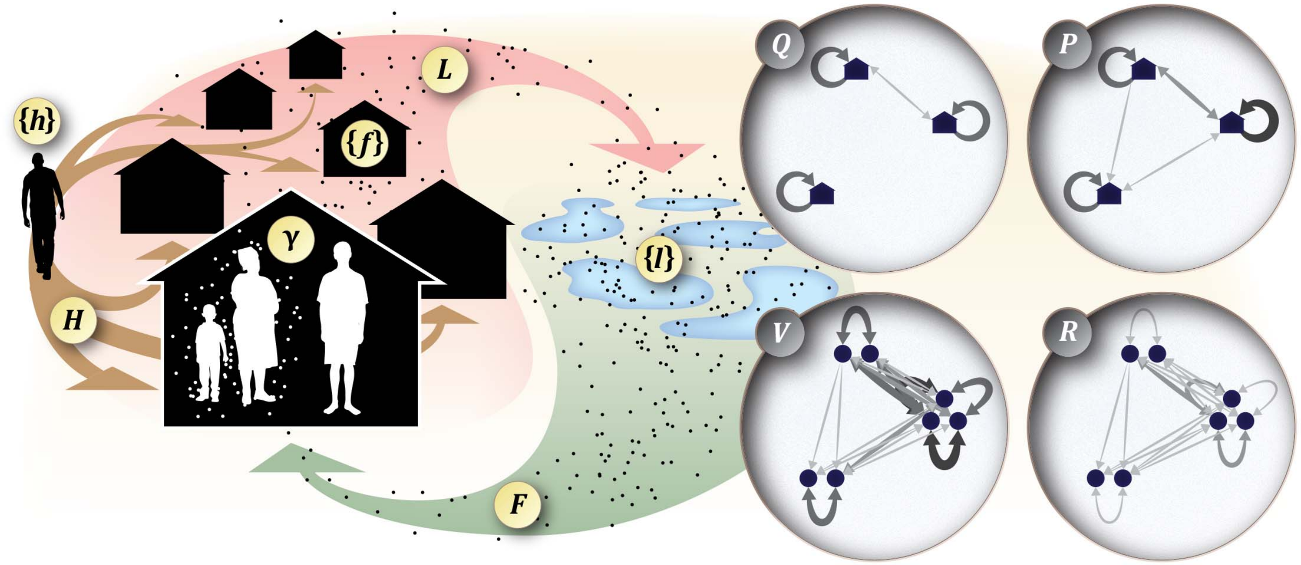

Figure 1. Model schematic. The model is specified on a continuous landscape with a point set of blood-feeding habitats, $\{f\}$, and a point set of aquatic habitats, $\{l\}$. The model is discrete in time with a time step equal to the length of the mosquito feeding cycle, in which mosquitoes take a blood meal, search for aquatic habitat (L, red arrow), lay eggs, and repeat the search for another blood meal (F, green arrow). Each host in $\{h\}$ allocates its time proportionally at multiple blood-feeding habitats (H, brown arrows). During a single feeding cycle, each mosquito present at a given blood-feeding habitat takes a single blood meal, the collection of which are distributed differentially on hosts according to the proportion of time each spends there and a quantity describing each host's biting suitability (γ). This model structure allows for the derivation of weighted, bidirectional networks that summarize pathogen dispersion among blood-feeding habitats (houses) or among hosts (circles). From this process-based description of transmission, it is possible to derive network summaries of pathogen dispersion. Pathogen dispersion by mosquitoes: Q describes how mosquitoes taking an infective blood meal at one blood-feeding habitat distribute secondary, potentially infectious bites at other blood-feeding habitats. Pathogen dispersion by hosts: P specifies the probability that a secondary bite on a human infected at one blood-feeding habitat takes place at some other blood-feeding habitat. Pathogen amplification: V gives the total number of secondary bites on a host arising from primary bites on another host in a single feeding cycle. Host infection: R contains the probabilities that a primary infection in one host will result in a secondary infection in some other host.
doi:10.1371/journal.pcbi.1003327.g001

the lattice. In either case, mosquito movement among the blood-feeding habitats is based on some description of mosquito behaviors relevant to the ecological needs dictated by a given species' natural history.

In the model, another set of points, denoted $\{l\}$, containing $|l|$ objects, describes the aquatic habitats where mosquito eggs are laid, develop into larvae, and then pupate before emerging as adults. At times, adult female mosquitoes make movement decisions based on the need for blood feeding, digestion and rest, sugar feeding, mating, egg laying, or to satisfy other biological needs. At a minimum, movements of epidemiological interest are those geared towards blood feeding and egg laying. Here we focus on the dispersion of mosquitoes (and of the pathogen by mosquitoes) based largely on matrices describing in probabilistic terms how mosquitoes move from aquatic habitats to blood-feeding habitats (F) and then vice versa to lay eggs (L). These matrices are derived from the co-distribution of the two point sets and mosquito search algorithms describing how mosquitoes locate and choose a particular blood-feeding habitat or a particular aquatic habitat. These matrices describe patterns of egg laying and blood feeding by adult mosquitoes as they move among aquatic habitats to lay eggs (FL) or among blood feeding habitats to blood feed (LF). The formulas suggest a close correspondence between movements for mosquito egg laying and movements for mosquito blood feeding and pathogen transmission.

The number of bites at each blood feeding habitat is related to these movement rules and to the productivity of each aquatic habitat, defined as the number of adult mosquitoes emerging from that habitat each day, Λ. Productivity depends on the number of eggs laid and the changes in larval survival in response to available food and crowding in each aquatic habitat, as well as other factors. The number of eggs laid by adult mosquitoes in each habitat is, in turn, related to the patterns of emergence and movement patterns of adult mosquitoes, generally after taking a blood meal. The dynamic coupling between blood feeding by adult mosquito populations and the ecology of aquatic mosquito populations is of great importance for mosquito population dynamics and pathogen transmission.

The location of aquatic habitats and ecology of immature mosquitoes can be highly variable (*e.g.*, due to changes in rainfall, temperature, resource availability, predation), but if the conditions remain constant, formulas describe the productivity at the steady state. Under these assumptions, it is possible to compute the number of bites occurring at each blood-feeding habitat, M. This is, in theory, the *in silico* analogue of the number of mosquitoes present at each blood-feeding habitat on each day and, therefore, proportional to the number that would be caught in a population monitoring program. For humans, it is thus related to the household biting rate that could be measured with pyrethroid spray catches, exit traps, CDC light traps, or by aspiration.

As a practical way of computing human biting rates (*i.e.*, the number of mosquito bites by vector species, per human, per day) from field data describing household biting rates, the number of mosquitoes caught is divided by the number of humans living in a house. In fact, some of those bites can occur on people who do not live in the house, and conversely some biting can occur on humans while they are at other people's houses. Similar arguments apply generally to other host populations. To complete the picture of heterogeneous biting, a description of host movement is required.

Movement behaviors of hosts are complex, with requirements to visit certain locations for sustenance or social interactions, for example. To avoid the specificity of those complexities, we define a set of hosts, $\{h\}$, and represent host movement simply by the proportion of an individual's time, on average, allocated at each blood-feeding habitat at times when mosquitoes are actively feeding, described by a matrix H. Hosts do not necessarily allocate their time at a location because there are or are not mosquitoes present, so the proportion of time a host allocates at all blood-feeding habitats could sum to less than one if it spends some of its time elsewhere.

The distribution of mosquito blood meals among all the hosts present is modeled as the confluence of mosquito and human movement leading to a set of potential encounters at a particular blood-feeding habitat. Complicated host behavioral responses, such as avoiding mosquitoes when their densities are high, can be simulated in this framework, but what matters for heterogeneous biting is the actual distribution of bites, which is determined in the model by a simple rule that allocates bites among hosts. A single number determines this rule, called the biting suitability score, denoted γ, which summarizes a large number of host factors (*e.g.*, body size, use of an ITN, wearing protective clothing, defensive behavior, etc.) that determine the proportion of bites that occur on each human at each location. A matrix, B, is derived that describes the expected number of bites occurring on each host at each blood-feeding habitat. Each row of B describes how mosquito blood feeding is allocated among humans at a particular place, and each column in B describes how many bites a particular human receives at every location. Heterogeneous biting is described by the normalized column sums that give a person's biting weight, w, which is the proportion of all the bites taken on each person.

In sum, heterogeneous biting is the product of the following: 1) mosquito population dynamics and movement leading to heterogeneity in the number of mosquitoes present at each blood feeding habitat; 2) the number of hosts sharing the risk at each blood feeding habitat and a rule that determines how the bites are distributed among them; and 3) the mobility of the blood meal hosts and their propensity to spend time at risk among many blood feeding habitats. Heterogeneous biting, in this framework, is represented in matrix form, emphasizing the difficulty of measuring heterogeneous biting in any simple way. The measures that can be used to estimate heterogeneous biting by catching mosquitoes at a place give a partial and useful snapshot of a more complicated process. This mathematical description outlines how it would be possible to integrate all the factors contributing to heterogeneous biting and give a full estimate.

Pathogen Dispersion

A pathogen's transmission through a population is typically thought of in terms of the total number of hosts infected by each infected mosquito, and vice versa [17]. In the Ross-Macdonald theory for mosquito-borne pathogen transmission, well-known quantities of this type include vectorial capacity and the basic reproductive number, R_0 [68,71]. Complementing these measures of potential transmission is a set of closely related field metrics measuring the intensity of biting and exposure including the human biting rate and the entomological inoculation rate [5,72]. Here, we use the framework to explore the richer dynamics of pathogen transmission and the patterns of dispersion among specific locations and individuals that give rise to the distributions of biting by describing probabilistic movement processes of individual mosquitoes, hosts, and pathogens structured by the locations of mosquito blood-feeding habitats. We use this framework to describe heterogeneous biting, pathogen dispersion, vaccine thresholds, the degree of mixing, and characteristic spatial scales of pathogen transmission.

The matrix descriptions of mosquito and human movement (above, Fig. 1) were also used to derive matrices describing

pathogen dispersion by mobile mosquitoes that account for mortality during pathogen incubation in the mosquito and all subsequent blood meals (Q), pathogen dispersion by mobile hosts (P), and dispersion through the full transmission cycle. The core of our analysis of pathogen transmission utilizes next-generation matrices that encode the networks we call V and R, corresponding to the classical concept of vectorial capacity and reproductive numbers. The matrix V contains the expected number of secondary bites on a host that comes from mosquitoes that took a primary bite on a given host during a single feeding cycle. The matrix R contains the probabilities that each one of the individual hosts will be infected by secondary infectious bites derived from any other given host. These matrices, as well as Q and P, effectively define weighted, bidirectional networks of expected pathogen transmission between locations or between individual hosts (Fig. 1). Summing across the rows of V and R yields vectors that we call $\vec{v}$ and $\vec{r}$. Rather than describing the expected numbers of secondary bites or infections between every pair of hosts, they contain the total numbers of expected secondary bites or infections arising from each primary (*i.e.*, reference) host. These vectors thus serve as literal, individual-level analogues of vectorial capacity and R_0 [71].

Spatial Scales of Transmission

Because our model formulation is inherently spatial, it presents a unique opportunity to answer questions about the spatial scales of pathogen dispersion. These scales, which define how localized transmission is, are directly relevant to a number of practical issues in the study and control of mosquito-borne pathogens. Examples include how big of an area must be sampled to estimate human biting rates, how large of an area must be sprayed to control an outbreak, the number of houses to visit for active case detection, and over what area must vaccines be distributed to effect sufficient herd immunity to attain local elimination? Answers a mathematical model provides to these questions will depend very much on its assumptions about spatial scales.

Distance Kernels

One very direct way to characterize the spatial scales of transmission under our framework is to compute distance kernels for any or all of the matrices we derived. These kernels are probability densities over space that describe how far away the events summarized by each matrix take place relative to where they originated. For example, the distance kernel, $k_Q(\delta)$, for the Q matrix describes the probability that, at some point in its life, a mosquito takes a blood meal a distance δ away from where it took a previous blood meal. The distance kernel, $k_S(\delta)$, for the S matrix (a spatial analogue of R) describes the probability that a secondary vertebrate host contracts the pathogen a distance δ away from where its corresponding primary host contracted the pathogen. These kernels can be specified either as a collection of kernels for individual hosts or locations, or they can be averaged over the population. And while they give a rich description of pathogen dispersion in space, they can also be used to give a simple and direct answer to the question of what the spatial scales of transmission are: the average distance, $\int \delta k_\cdot(\delta)d\delta$, between where consecutive events take place. Fig. 2 contains examples of these kernels for a simulated landscape.

Mixing within Patches

Another way to define the spatial scales of transmission is to consider mixing within a "patch" of a given size. Patch, or metapopulation, models of mosquito-borne pathogen transmission assume that interactions within each patch are random and that movement between patches by mosquitoes and hosts occurs at various rates. To begin to address the issue of the spatial scales of transmission in this patch context, we must first define two related but distinct concepts: heterogeneity and mixing. In a patch that is well mixed but displays high heterogeneity, mosquitoes take more bites on some hosts than others, but the identities of hosts that receive consecutive bites from a mosquito are uncorrelated. In a patch that is poorly mixed, there are in some sense "partnerships" between certain mosquitoes and certain hosts, whereby a mosquito that has bitten one host is more likely to then bite certain hosts than others.

Both of these properties likely manifest in natural systems, but they derive from different processes and have different implications for model structure and dynamics. What we term as heterogeneity is typically thought to derive from preferential biting by mosquitoes on certain hosts, but it can also be impacted by the extent to which people spend time in places with lots of mosquitoes and how many other people there are to dilute their risk of biting. Regardless of its causes, this property is relatively easy to include in a patch model (*e.g.*, [21,68]). Mixing, on the other hand, is fundamentally a "distance" concept. For example, mosquitoes that bite one host are more likely to take their next blood meal on that host, its cohabitants, or other hosts nearby, than they are to subsequently encounter a host that is far away. The concept of distance may also apply to proximity on a social network, as well [50]. Patch models universally assume that interactions within a patch are perfectly mixed, so using a more reductionist framework like ours provides an opportunity to test the validity of this assumption at different scales. Intuitively, one would expect small patches with frequent interactions among few actors to be well mixed relative to increasingly large patches with less frequent interactions among a larger pool of actors. Characterizing this relationship and identifying at what scale patches tend to become poorly mixed is precisely our goal.

To quantify these properties we need mathematical definitions of the concepts of a patch, a description of transmission within a patch, and a way to separate heterogeneity and mixing. This procedure could potentially be applied to any of our matrices, but we focus on the S matrix, which is most relevant to the spatial scales of transmission among hosts. This matrix is defined on the set of blood-feeding habitats, so in this case a patch could be defined as any subset $p \subseteq \{f\}$. Because here we are concerned only with dynamics within a patch, we focus on the matrix S_p, which is obtained by deleting all rows and columns of S that correspond to blood-feeding habitats outside of the focal patch p. In the context of the S matrix, heterogeneity manifests as uneven *total contributions* of transmission from hosts at different constituent locations, whereas mixing manifests as an uneven *distribution* of secondary infections arising from hosts at each constituent location. Mathematically then, heterogeneity in a patch is concerned with the sums across the rows, and mixing is concerned with the normalized vectors from each row. The extrema of mixing are the scenarios in which 1) all values of the normalized rows are equal (perfectly mixed), and 2) one entry of each normalized row equals 1 and the rest equal 0 (no mixing). A familiar measure with extrema corresponding to these scenarios, and that can be used to quantify the myriad possibilities in between, is evenness [73]. Our proposed measure of mixing within a patch therefore uses the matrix $S_p{}'$, whose rows equal the normalized rows of S_p, to define

$$e(p) = \frac{\sum_{i \in p} \sum_{j \in p} S_p{}'[i,j] \ln S_p{}'[i,j]}{|p| \ln |p|^{-2}} \tag{1}$$

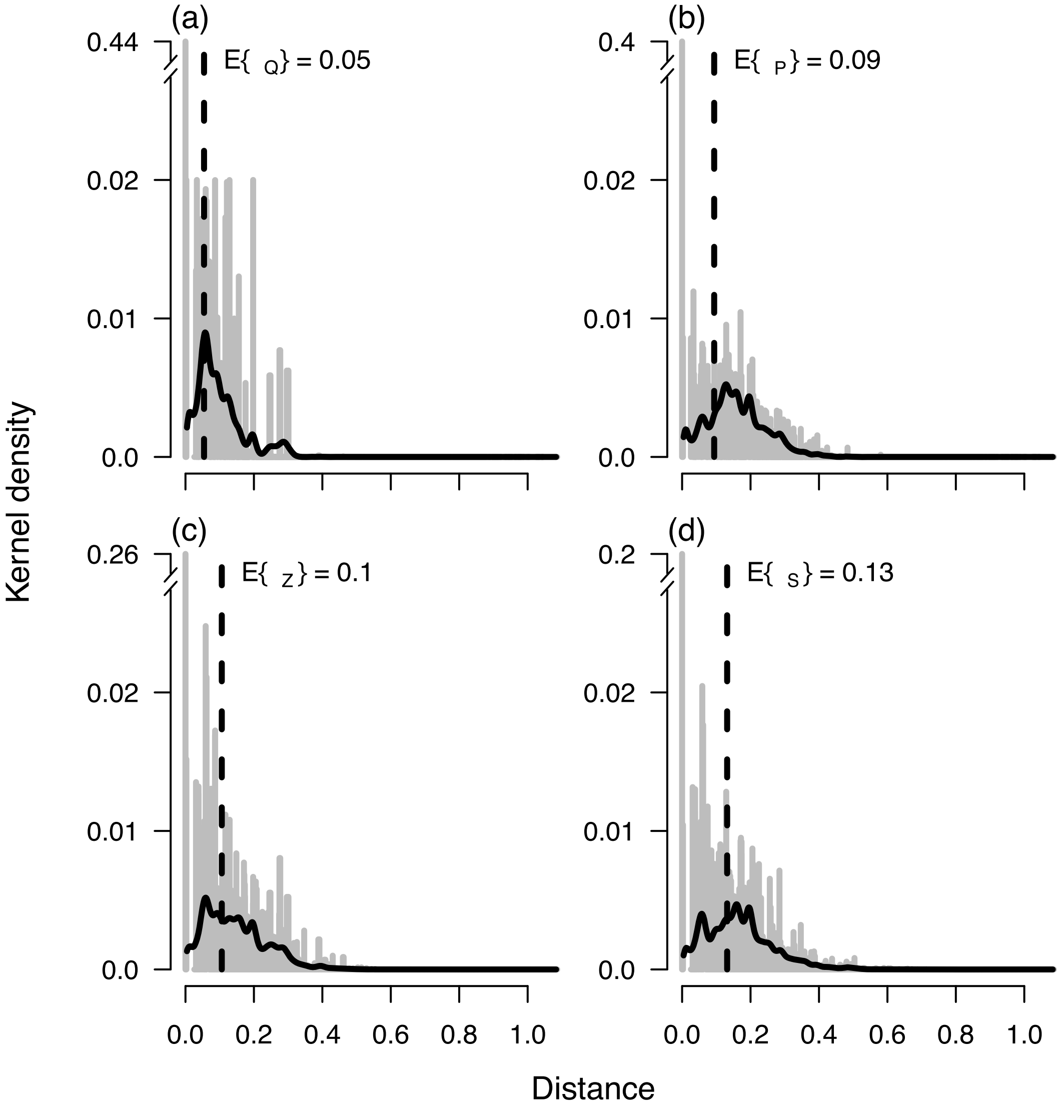

Figure 2. Spatial kernels. Panels correspond to the matrices that summarize pathogen dispersion by mosquitoes (a), pathogen dispersion by vertebrate hosts (b), pathogen dispersion through both species (c), and the spread of secondary host infections (d). Gray histograms show the empirical densities of each matrix's weighting at different distances, and black curves show a smoothed version of these data. Dashed lines show the average distance at which the events described by each matrix take place and therefore represent one way of defining the spatial scales of transmission with a single number. For example, the dashed line in (d) indicates that, on average, mosquito bites conferring a secondary host infection occur a distance of $\mathrm{E}\{\delta_S\}=0.13$ away from where the corresponding primary host transmitted the pathogen to a mosquito.
doi:10.1371/journal.pcbi.1003327.g002

which provides a quantitative basis for comparing the degree of mixing in different patches.

To address the issue of the spatial scales of transmission, we chose patches of different sizes and examined the relationship between mixing and a patch's size and spatial extent. For a given set of blood-feeing habitats, $\{f\}$, there are any number of reasonable ways to choose patches for this analysis. The algorithm that we adopted involves starting from a full set $\{f\}$ and agglomerating groups of locations based on the distance between their centroids, doing so successively until all f are grouped together in a single patch (Figs. 3, 4). Given definitions of different patches representing the full spectrum of patch size and spatial extent, we then evaluated the evenness of mixing, $e(p)$, on each of them. As expected, there is a clear positive relationship between patch size (and spatial extent) and poor mixing (Fig. 3). One way to make use of this relationship to define the spatial scales of transmission is to select a threshold value of the evenness of mixing and select the set of patches whose $e(p)$ correspond to that threshold. Regardless of the specific value chosen for this threshold, it is nonetheless a useful procedure for establishing how mixing varies across spatial scales and for comparing this relationship across different ecological contexts (*e.g.*, columns of Fig. 3).

Although mixing is expected to vary across spatial scales and thus be most informative for an effort to identify the spatial scales of transmission, it is also likely that there is epidemiologically relevant variation in the heterogeneity displayed within different patches. To explore this variation, we took the same hierarchy of patches from the analysis of mixing and, for each patch, evaluated the coefficient of variation of sums across each row of S_p (Fig. 4). In doing so, we find that small, spatially restricted patches display a great deal of variation in the extent of within-patch heterogeneity

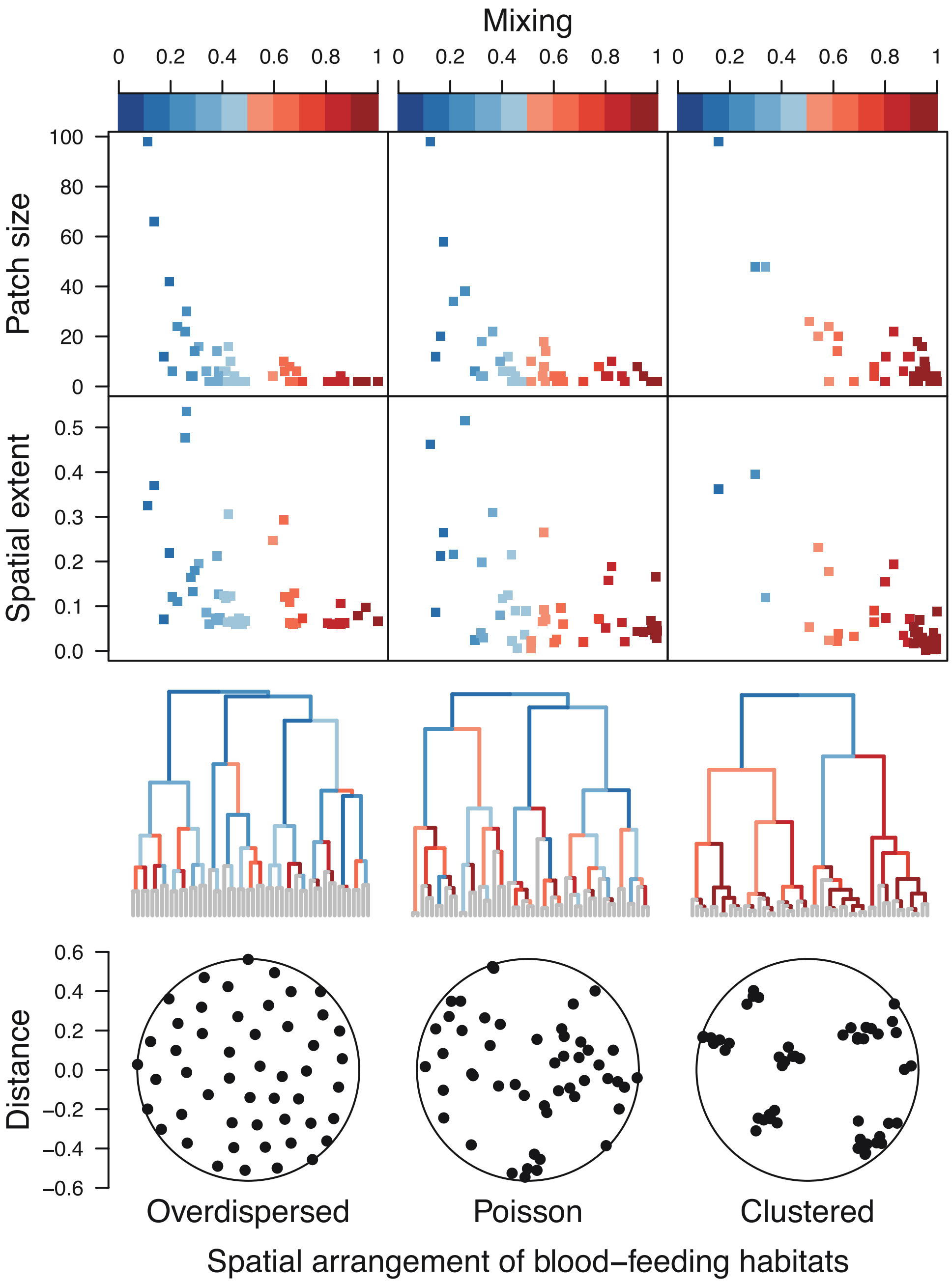

Figure 3. Mixing across scales. Evenness of mixing of secondary infections within subsets of blood-feeding habitats under different assumptions about their spatial arrangement. Phylograms are structured from bottom (depiction of spatial arrangement of blood feeding habitats) to top (by spatial extent and patch size) such that nearby blood-feeding habitats are grouped together, nearby groups combine to form larger groups, and so on, until all blood-feeding habitats are grouped together. Colors on the branches of the phylograms show the evenness of mixing in the diagonal submatrix of S corresponding to blood-feeding habitats that comprise each cluster.
doi:10.1371/journal.pcbi.1003327.g003

and that the largest, most expansive patches tend to have moderate to high heterogeneity. Thus, there is potential for heterogeneity at all spatial scales, but it appears to be increasingly visible at larger scales that involve aggregation over more individuals and larger areas.

Dynamics across Scales

Given these ways of characterizing the spatial scales of transmission, a natural question that arises is how epidemiological dynamics and the impacts of control measures vary across systems with different characteristic scales of transmission or across multiple spatial scales in a single system. Because all models must make one assumption or another about the relationship between heterogeneity, mixing, and the spatial scales of transmission, the answer to this question has direct implications for the accuracy of quantitative predictions flowing from them. Below, we use our modeling framework to illustrate that models with differing assumptions about heterogeneity and mixing make vastly different quantitative predictions about epidemic dynamics and R_0-based vaccination thresholds.

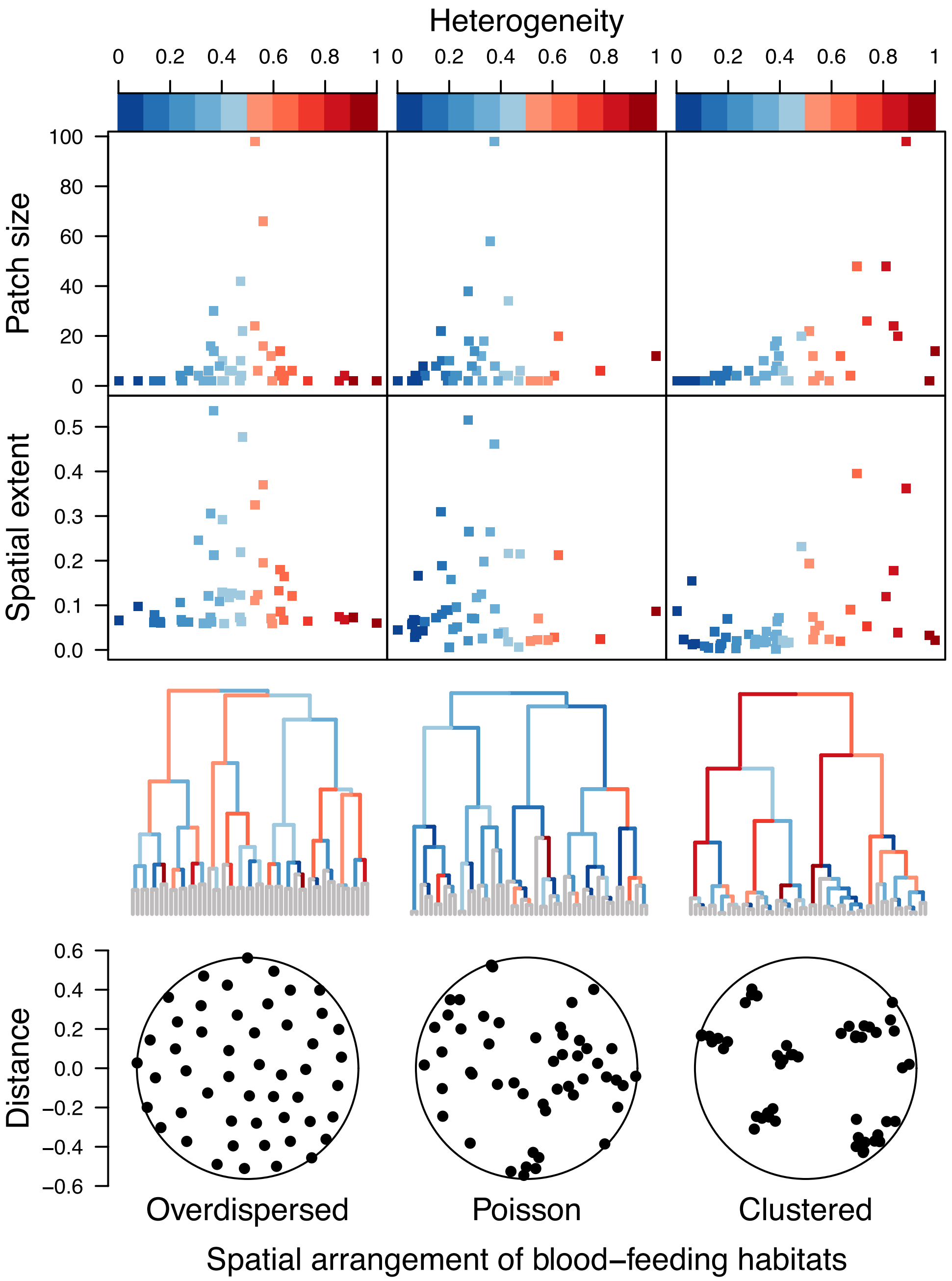

Figure 4. Heterogeneity across scales. Heterogeneity of outgoing secondary infections from subsets of blood-feeding habitats under different assumptions about their spatial arrangement. Phylograms are structured from bottom (depiction of spatial arrangement of blood feeding habitats) to top (by spatial extent and patch size) such that nearby blood-feeding habitats are grouped together, nearby groups combine to form larger groups, and so on, until all blood-feeding habitats are grouped together. Colors on the branches of the phylograms show the coefficient of variation of the sums across the rows of the diagonal submatrix of S corresponding to blood-feeding habitats that comprise each cluster.
doi:10.1371/journal.pcbi.1003327.g004

Epidemic Dynamics

Although the concepts of heterogeneity and mixing can be disentangled in summary matrices such as R and S, it is less straightforward to separate them in the full model because heterogeneous biting and contacts tend to go hand in hand with localized movement and poor mixing. It is nonetheless instructive to consider the different dynamics exhibited by models at both extremes: *i.e.*, one with localized movement and heterogeneous exposure, and one with uniform movement and exposure.

Simulating 100 realizations of an epidemic with our stochastic model, we see that poor mixing and heterogeneity have considerable impacts on the number of infected hosts over time (Fig. 5c). Most notably, an epidemic in a heterogeneous, poorly mixed system progresses more slowly, has a more variable progression over time, and ultimately infects fewer hosts than would be suggested by a model that assumes uniform, well-mixed interactions at the same spatial scale. A clue to understanding why these different scenarios exhibit different dynamics can be found by examining the cumulative exposure of individual hosts in the

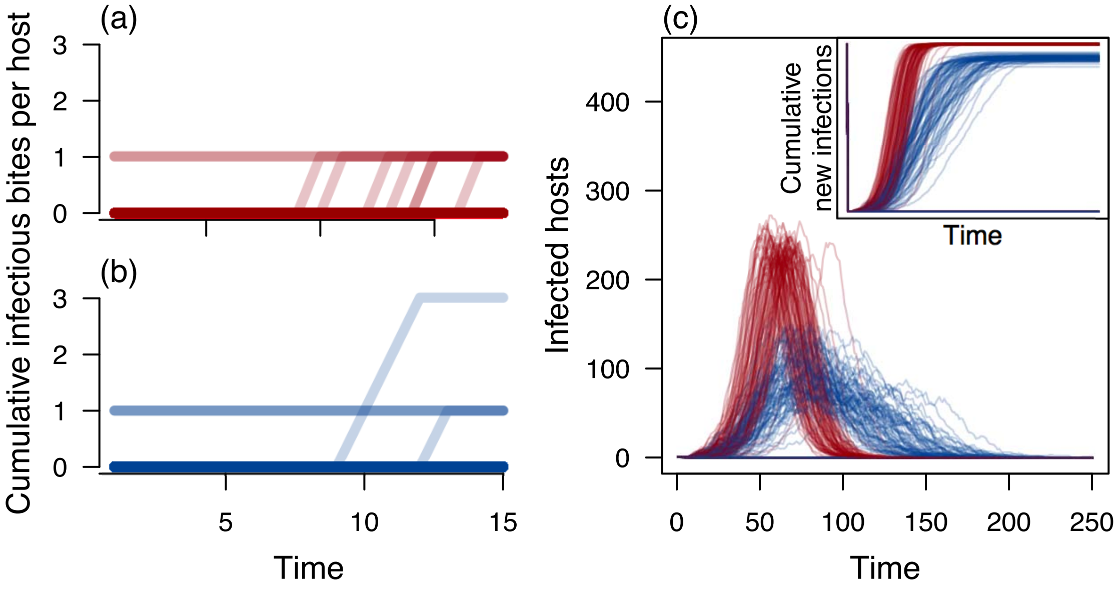

Figure 5. Epidemic dynamics across scales. When mosquito and host movement are both well mixed (a), each infectious bite originating from a single primary host is made on a unique secondary host. When mosquito and host movement are both poorly mixed (b), some hosts receive multiple infectious bites. Under these different scenarios about movement, epidemics originating in hosts with equal r_k unfold much differently. Pathogen spread through a well-mixed population is quick, consistent, and complete (red in c), whereas pathogen spread through poorly-mixed populations is slower, more variable, and does not infect the entire host population (blue in c).
doi:10.1371/journal.pcbi.1003327.g005

population over time. In a model with well-mixed interactions and uniform exposures, each infectious bite tends to occur on a different individual host (Fig. 5b). In a model that accounts for heterogeneous exposure and poor mixing, however, multiple infectious bites tend to consistently fall on the same subset of hosts (Fig. 5a), meaning that some bites are redundant and that there are fewer new infections over time. Any model that aggregates hosts and mosquitoes into a uniform, well-mixed whole at an inappropriately large spatial scale has the potential to exhibit dynamics that are biased in this way.

R_0 and Vaccination Thresholds

At any given spatial scale, there are at least three general factors acknowledged in our modeling framework that have a direct impact on estimates of the basic reproductive number, R_0: heterogeneity, mixing, and finite numbers of hosts. The effects of these factors can be examined directly under our framework by manipulating the summary matrix R.

For this comparison, we first simulated a transmission landscape and calculated R_0 as the dominant eigenvalue of R, which serves as the benchmark against which all other methods for calculating R_0 a priori (*i.e.*, based on parameters and a formula) can be compared. Note that this formulation allows for all three of the factors affecting R_0 listed above. The other methods for calculating R_0 that we consider include the Ross-Macdonald formula (reviewed in [71]), an approximation accounting for heterogeneous biting first applied to mosquito-borne pathogens by [21], an adjustment to the approximation by [21] accounting for finite-host numbers [68], and what we refer to as an "unbounded next-generation approach" that accounts for heterogeneity and poor mixing but not finite host numbers [58]. The relative values of R_0 calculated by these different methods differ depending on the overall intensity of transmission, which can vary depending on mosquito density, mosquito lifespan, the pathogen incubation period in mosquitoes, and how often mosquitoes blood feed. As a proxy for the rest of these situations, we examined two scenarios with relatively high and low mosquito survival (low transmission and high transmission, respectively).

In a low-transmission context, R_0 is greatest under the unbounded next-generation approach [21] and somewhat less for our approach based on the R matrix. Values of R_0 are then progressively less for the [21] approximation, the [68] formula with heterogeneity and finite host numbers, and finally for the Ross-Macdonald formula (Table 1). In a high-transmission context, however, the methods by [58], [21], and Ross and Macdonald all lead to values of R_0 that exceed its value under our model (Table 1). Together, these patterns suggest that the inclusion of increasingly complex heterogeneities in otherwise equivalent systems increase a priori estimates of R_0, but that all such increases are tempered by finite host numbers in increasingly intense transmission contexts. Furthermore, the extent to which these effects of finite host numbers manifest on estimates of R_0 depends on the presumed spatial scale of transmission. Rather than depending on host population size per se (as might be interpreted based on a literal interpretation of [68]), the effects of finite host numbers manifest at the *individual* level. What really matters, then, is how many potentially infectious bites are concentrated on certain individuals. For example, in the most poorly mixed system possible, mosquitoes would show biting fidelity on individual hosts, never allowing for the possibility of any secondary infections ($R_0=0$). Were infectious bites to become distributed on larger and larger numbers of hosts, the potential for secondary infections would grow and so would R_0. This basic reproductive rate then clearly depends very much on the extent of mixing in a population, which is a consequence of a relatively fine spatial scale of transmission vis-à-vis mosquito movement, host movement, and the spatial distributions of mosquitoes and vertebrate hosts. Because vaccination proportions, p, necessary to prevent pathogen invasion or to achieve local elimination are often guided by estimates of R_0 and the fundamental relationship

Table 1. Basic reproductive numbers (R_0) and vaccination thresholds (p) calculated by methods that variously account for heterogeneity (H), poor mixing (M), and finite host numbers (F), in both low- and high-transmission contexts in a randomly simulated population.

				Low transmission			High transmission		
**Method**	**H**	**M**	**F**	R_0/R_0'	R_0	p	R_0/R_0'	R_0	p
Ross-Macdonald	–	–	–	0.61	1.19	0.16	1.29	33.77	0.97
Favier *et al.* 2006	–	–	–	0.72	1.41	0.29	0.07	1.85	0.46
Dye & Hasibeder 1986	✓	–	–	0.81	1.57	0.37	1.55	40.56	0.98
Smith *et al.* 2007	✓	–	✓	0.79	1.54	0.35	1.48	38.77	0.97
Hasibeder & Dye 1988	✓	✓	–	1.09	2.11	0.53	1.86	48.70	0.98
present study	✓	✓	✓	1	1.93	0.48	1	26.10	0.96

Because the value of R_0 based on the R matrix (denoted here as R_0') is the most complete description of the underlying transmission process, we also list the ratio of all other values to it (R_0/R_0'). All methods compute R_0 based solely on parameter values of the model, except Favier *et al.* 2006, which use both a subset of model parameters and an empirical estimate of the force of infection based on simulated epidemic data.
doi:10.1371/journal.pcbi.1003327.t001

$p > 1-1/R_0$, such systematic differences in a priori estimates of R_0 due to poor mixing beyond some characteristic spatial scale are all subject to analogous systematic errors in the estimation of vaccination coverage levels (Table 1).

Another approach to estimating R_0 is to do so based on an empirical estimate of the force of infection at the onset of an epidemic. As an example of this general approach, we compute the estimate of R_0 derived by [74] (eq. 6), which was formulated specifically for application to mosquito-borne pathogens. Their formula depends on an empirical estimate of the force of infection, as well as the duration of infectiousness and pathogen incubation periods in hosts and mosquitoes. Importantly, the mathematical model underlying their formula is one much like the Ross-Macdonald model in that it assumes uniform exposure, perfect mixing, and does not account for the fact that each host can contract at most one secondary infection. Taking the average estimate of force of infection from 100 simulated epidemics and applying it to the formula of [74], we find that this estimate of R_0 is consistently lower than all a priori estimates of R_0, regardless of the intensity of transmission (Table 1). This disparity between an empirical estimate based on data from a truly heterogeneous, poorly mixed, and finite population and the true value of R_0 in that population (*i.e.*, our estimate based on the R matrix) can be accounted for by the fact that a model that assumes uniform exposure, perfect mixing, and an infinite supply of susceptible hosts would require much less intense transmission to produce similar dynamics (as can be intuited via Fig. 5). Were such systematically low estimates of R_0 used to guide planning for vaccine deployment, an inadequate proportion of the population would be vaccinated and a pathogen would be more likely to invade or persist (compare p in Table 1).

Discussion

Many mathematical models of mosquito-borne pathogen transmission have focused on infection and the factors that determine the intensity of transmission at the expense of details of how pathogens disperse through populations [17]. For the time being, we have made the opposite tradeoff by eschewing many possible pathogen-specific details of infection dynamics and focusing instead on ecological aspects of dispersion common to transmission of all mosquito-borne pathogens. A central theme of this perspective is that, inasmuch as movement patterns of mosquitoes and humans are limited to relatively few mosquito blood-feeding habitats, repeated transmission events within certain groups of hosts and mosquitoes break the standard assumption of well-mixed encounters at the population level. In the likely event that movement is spatially constrained (*e.g.*, in mosquitoes by energy expenditure and mortality risk, or in humans by convenience and cost), this effect of poor mixing gives rise to characteristic spatial scales smaller than the area over which mosquito and host populations are distributed as a whole. Using information about movement patterns and spatial distributions of mosquitoes and hosts, our model allows for these scales to be quantified in at least two ways, based on (1) spatial kernels of consecutive transmission events, and (2) patterns of mixing across scales of aggregation. We also demonstrate the consequences of ignoring poor mixing and the characteristic scales of transmission, which include misinterpreting epidemic patterns and biasing estimates of the basic reproductive number and critical vaccination threshold.

The effects of heterogeneity, poor mixing, and finite host numbers on the transmission of mosquito-borne pathogens have been described and modeled before (*e.g.*, [21,56–58,68,75]). Yet

the simultaneous linkage of all three of these factors, which is important for more than the sake of completeness, has heretofore not been made. Two foundational theoretical papers by Dye and Hasibeder [21,58] established that heterogeneous biting and poor mixing lead to increases in R_0, which make pathogen invasion more likely and elimination more difficult. Later, Smith *et al.* [68] noted that increases in R_0 due to heterogeneous biting are limited by the finite number of hosts on which those bites can be distributed, implying that R_0 may in many cases be lower than previous theory would suggest. By combining all three of these factors, we find that the truth is more complicated and likely somewhere in between. That is, heterogeneity and poor mixing do increase a pathogen's ability to invade and persist in a population, but these effects are limited by the number of hosts in an area, the size of which is determined by ecological factors that define the characteristic spatial scales of transmission. Similar to issues that arise in statistical inference based on network models [76], inferences about R_0 or other measures of transmission made from data under mass-action assumptions are biased to an unknown degree from their true values, which depend on the extent of poor mixing. Perhaps even more troublingly, a priori estimates of vectorial capacity and R_0 made by plugging values of component parameters into classic formulae are biased to an unknown degree and in unpredictable directions. The theoretical developments we have made represent an important step in identifying and addressing these problems, but more work to empirically quantify fine-scale heterogeneity and patterns of mixing in ecologically diverse systems is needed.

Another feature of much existing theory that may have limited its adoption or application is the lack of a clear connection to underlying biological mechanisms. Rather than address heterogeneous biting or exposure in a generic way, our model contains many of the biological elements that likely contribute to heterogeneity in real systems. The dominant mechanism for heterogeneous transmission promoted to date has been variation in factors such as body size or age of hosts. Although our results do not preclude the importance of these factors, they underscore that patterns of host and mosquito movement could also be important sources of heterogeneous transmission, too. In our model, the foremost requirement to obtain movement-based heterogeneity is that an individual host allocates its time at only a subset of locations where blood feeding occurs. This rather basic assumption means that some blood-feeding habitats will be frequented more often and by more hosts, whereas others will be visited less often. Patterns of spatial variation in mosquito density and movement of mosquitoes and hosts will then jointly determine the potential for any individual host or mosquito to transmit a pathogen to some subset of the rest of the population. Although working out such fine-scale details of heterogeneity and transmission in real systems will be a formidable challenge, our framework takes an important step by laying down the mathematical foundation with which measurable properties of individual vertebrate hosts and the locations they frequent can be translated into transmission potential. Likewise, even if such consistent variation in these characteristics cannot be assessed at an individual level, their impacts on patterns of spatial variation in transmission can be assessed with our framework at whatever scale data are available.

These advances in theory for mosquito-borne pathogen transmission have direct implications for policies regarding the deployment of control measures in these systems. In particular, vaccines have received an especially high level of interest from modelers recently [17,77,78] due to the late-phase trials of vaccines for dengue and malaria. Comparison of our model with some routinely applied to vaccination shows that disregard of heterogeneity, poor mixing, and finite host numbers may lead to incorrect estimates of coverage levels necessary to achieve herd immunity. Even worse, we show here that whether these models underestimate or overestimate necessary coverage levels is not always consistent, and therefore not predictable or easily correctable, across contexts. On the other hand, basing these predictions on empirical estimates of the force of infection, in combination with models based on assumptions of uniform exposure and perfect mixing, leads to a consistent bias of always underestimating necessary vaccination coverage levels. Relative to existing methods, our modeling framework also has the advantage of enabling the assessment of targeting vaccine delivery to individuals based on measurable properties, such as where they live or how extensive their social network is. In addition, the ability to calculate spatial kernels of pathogen transmission has direct applicability to determination of the coverage areas for mosquito spraying in response to active cases.

Common criticisms of individual-based models—which ours is not limited to, but is at its core—include the difficulty of parameterizing them and their analytical intractability. In practice, however, our framework provides at least some analytical insight and is clear about what parameters must be specified and the scales at which they should be measured. Specifically, the main parameters that must be specified for our model, beyond those that must be defined for any comparable model, are the spatial distribution of habitats, hosts, and mosquitoes, and movement patterns among those habitats. For many applications, the coordinates of habitats can be informed by GIS or remote sensing data [63,79]. Data pertaining to the spatial distributions of mosquitoes and vertebrate hosts are becoming available at increasingly fine scales [80,81], and plausible summaries of individual movement patterns linking those populations could be derived with a combination of behavioral algorithms [82] and data from movement studies [83,84]. Even if reasonable estimates of individual- or household-level parameters are not available, the model is flexible enough to permit specification of patches defined on whatever scale data are available or can be reasonably imputed. At any of these scales, the matrices we have derived can be calculated to provide more detailed and biologically meaningful alternatives to classic scalar metrics, such as vectorial capacity and R_0, which ignore heterogeneity, mixing, and their sensitivity to finite vertebrate host numbers at the characteristic spatial scales of transmission. Such fine-scale analytical tools provide an important link between known theoretical insights and complex simulation-based models that are becoming increasingly applied to important, policy-relevant problems.

Methods

Mosquito blood meals are the focal event in pathogen transmission, and this model is based on a mathematical construct describing where mosquito-host encounters occur. Many mosquitoes have well-defined haunts and blood-feeding habits, such that blood feeding tends to occur in places that can be reasonably well characterized. These are rigorously described as a set of points, denoted $\{f\}$, containing $|f|$ objects, called *blood-feeding habitats*. One way of defining $\{f\}$ could be to consider the collection of homes in a city, which might be appropriate for human populations afflicted by malaria or dengue. Another, more flexible option would be to impose a lattice over an area and aggregate blood-feeding that takes place nearest to each point on the lattice. In either case, mosquito movement among the blood-feeding habitats is based on some description of mosquito behaviors relevant to the ecological

needs dictated by a given species' natural history. At times, adult female mosquitoes will make movement decisions based on the need for blood feeding, digestion and rest, sugar feeding, mating, egg laying, or other factors. At a minimum, movements of epidemiological interest are those geared towards blood feeding and egg laying. Movement behaviors of hosts are also complex, with requirements to visit certain locations for sustenance or social interactions, for example. To avoid the specificity of those complexities, we represent host movement simply by the proportion of an individual's time, on average, allocated at each blood-feeding habitat. Hosts do not necessarily allocate their time at a location because there are or are not mosquitoes present, so the proportion of time a host allocates at all blood-feeding habitats could sum to less than one if it spends some of its time elsewhere. The way these assumptions manifest mathematically and their relevance to transmission dynamics are discussed in detail in the following sections.

Mosquito Movement

In our model, adult mosquitoes emerge from aquatic habitats, which are contained by a set of spatially referenced points $\{l\}$ containing $|l|$ objects. After they emerge, mosquitoes alternate moving between aquatic habitats and blood-feeding habitats. Every adult female mosquito that survives a pair of consecutive moves is assumed to lay eggs and blood feed once within a fixed interval corresponding to the length of the feeding cycle. Departures from these assumptions will vary in kind and degree for different mosquito species, but these core assumptions nonetheless constitute general features of the mosquito life cycle. With respect to our model, $\{f\}$ and $\{l\}$ together define a template for mosquito movement, and mosquito dispersal on these sets can be described concisely as a random walk on a weighted, bidirectional, bipartite graph.

Such random walks by mosquitoes are described by two matrices, one, an $|l|$-by-$|f|$ matrix F, describing mosquito movement to find a vertebrate host and feed on blood and the other, an $|f|$-by-$|l|$ matrix L, describing mosquito movement to find aquatic habitat and lay eggs. Other mosquito needs, such as finding mates, digestion and resting, and sugar feeding, are assumed to occur somewhere along the way and are not modeled explicitly. Let an element $F_{i,j}$ in F denote the proportion of mosquitoes that start from the ith aquatic habitat and end at the jth blood-feeding habitat with a successful blood meal. Similarly, let an element $L_{i,j}$ in L denote the proportion of mosquitoes that start from the ith blood feeding habitat and end at the jth aquatic habitat after laying eggs. Elements of F and L thus contain information about movement and mortality, with the sum across each row of F and L giving the mortality of mosquitoes between leaving one habitat and reaching any habitat of the other type. Mortality risks at different habitats could be quite variable, and survival during each flight could depend in some way on the distance traveled, type of habitat traversed, climatic conditions, or other factors.

Host Movement

For the purposes of transmission, host movement can be characterized by the proportional allocation of each host's time at each blood-feeding habitat and by the distribution of bites among the hosts that frequent each blood-feeding habitat. First, let $\{h\}$ denote a set of $|h|$ hosts, which remains fixed because we do not consider host births or deaths over the relatively short timescale of interest here. Then let each element $H_{i,j}$ of an $|h|$-by-$|f|$ matrix H denote the fraction of time that the ith host spends at the jth blood-feeding habitat. In the matrix H, each row thus represents a host's proportional allocation of time at the different blood-feeding habitats. Hosts can also spend some of their time at places outside the set of blood-feeding habitats, implying that $\sum_j h_{i,j} \leq 1$.

The number of bites a host receives depends not only on how it allocates its time at different locations, but also on how attractive mosquitoes find that host to be relative to other hosts at those locations and how effective a host's avoidance or defensive behaviors are. To take individual factors that affect biting attractiveness and avoidance or defensive behaviors into account, we assign each host a biting suitability score γ_i. The normalized values of those scores for all hosts at a given location give the probabilities that a mosquito at that location will bite each of those hosts. Given that H allows for different hosts to spend different proportions of their time at different blood-feeding habitats, the distribution of bites on hosts at each blood-feeding habitat must be jointly determined by host time allocation and biting suitability. Mathematically, bites at blood-feeding habitat i are distributed on hosts according to the vector

$$U_{i,\cdot} = (1-\psi_i)\frac{\gamma \cdot h_{\cdot,i}}{\sum_j \gamma_j h_{j,i}}, \tag{2}$$

where ψ_i is the proportion of bites taken on the focal host species at i. Collating these vectors for all blood-feeding habitats yields an $|f|$-by-$|h|$ matrix U.

Stochastic Transmission Dynamics

Having described mosquito movement with L and F, host movement with H, and the distribution of bites on individual hosts with U, it is now possible to layer a model of pathogen transmission on top of this framework. Here we specify a stochastic, individual-based SEIR model of host infection dynamics (susceptible, exposed, infected, recovered) and an SEI model of mosquito infection dynamics, although our framework is also capable of accommodating other types of infection dynamics. We describe an equivalent deterministic model that aggregates hosts and blood-feeding habitats in Text S1.

The infection status of host i is described by the ith entries of binary vectors S, E_e, I_i, and R. Infected hosts progress through each stage $e \in 1,\ldots,\sigma$, where σ is an integer number of feeding cycles that specifies the duration of pathogen latency in hosts. Infectious hosts progress through stages $i \in 1,\ldots,\rho_{max}$ until they recover at some time specified by a failure distribution

$$\rho_{\text{fail}}(i) = \Pr\{\text{recovery at } i \mid \text{still infectious after } i-1\} \tag{3a}$$

$$= \frac{\Pr\{\text{recovery at } i \cap \text{still infectious after } i-1\}}{\Pr\{\text{still infectious after } i-1\}} \tag{3b}$$

$$= \frac{\rho_{\text{pmf}}(i)}{1-\rho_{\text{cdf}}(i-1)}, \tag{3c}$$

which defines the probability of recovering after i feeding cycles conditional on still being infectious then. The probability mass function $\rho_{\text{pmf}}(i)$ and cumulative distribution function $\rho_{\text{cdf}}(i)$ define the failure distribution, and the mean duration of infectiousness is $\bar{\rho}$. Mosquito infection dynamics are modeled by partitioning adult female abundance, M, into vectors of length $|f|$ for each infection state, S', E'_e, and I' for $e \in 1,\ldots,\tau$, where τ is an integer number of

feeding cycles that specifies the duration of pathogen latency in mosquitoes. The other parameters relevant to transmission include the proportion b of infectious mosquito bites that cause a host infection and the proportion c of bites on infectious hosts that infect a mosquito. These quantities combine in the following way to completely specify the stochastic, individual-based dynamics for hosts,

$$S(t+1)=\text{Bernoulli}\left(S(t),(1-b)^{\sum_{\{f\}}\text{Multinomial}(I'(t),U)}\right) \quad (4a)$$

$$E_0(t+1)=S(t)-S(t+1) \quad (4b)$$

$$E_e(t+1)=E_{e-1}(t) \quad (4c)$$

$$I_0(t+1)=E_{\sigma-1}(t) \quad (4d)$$

$$I_i(t+1)=\text{Bernoulli}(I_{i-1}(t),1-\rho_{\text{fail}}(i-1)) \quad (4e)$$

$$R(t+1)=R(t)+\left(\sum I_i(t)+E_{\sigma-1}(t)-\sum I_i(t+1)\right), \quad (4f)$$

and for mosquitoes,

$$S'(t+1)=\text{Multinomial}(A_{\xi}(t+1),F)+\text{Multinomial}(S''(t),LF) \quad (5a)$$

$$S''(t+1)=S'(t+1)-\text{Binomial}(\text{Multinomial}(S'(t+1),U),cI(t)) \quad (5b)$$

$$E'_0(t+1)=S'(t+1)-S''(t+1) \quad (5c)$$

$$E'_e(t+1)=\text{Multinomial}(E'_{e-1}(t),LF) \quad (5d)$$

$$I'(t+1)=\text{Multinomial}(I'(t)+E'_{\tau-1}(t),LF), \quad (5e)$$

where A_{ξ} is a vector containing the number of new adult females emerging from $\{l\}$. The Bernoulli, Binomial, and Multinomial functions generate random numbers from those distributions with the supplied parameters. Random numbers are drawn independently across the vectors or matrices of parameters supplied, such that the dimensions of the vectors produced by the random-number functions balance with the dimensions of other terms in those equations.

Although this formulation emphasizes heterogeneity and stochasticity in movement, biting, and host recovery, it is possible to extend the model to account for several other factors. For example, some mosquito species are known to often take multiple blood meals between successive egg-laying events [85], yet eqq. (4) and (5) only allow for one blood meal per mosquito per gonotrophic cycle. If, on the other hand, mosquitoes take an average of β blood meals per gonotrophic cycle, this could be incorporated into transmission dynamics by multiplying $I'(t)$ by β in eq. (4a) and by replacing $cI(t)$ with $1-\exp(-c\beta I(t))$ in eq. (5b). Other factors that could be incorporated into a more refined version of the model for specific applications include individual-level heterogeneity in the probability that hosts confer infections to mosquitoes that bite them, among others. Factors relevant to transmission could also display heterogeneity at broader spatial scales or over time, such as temperature-dependent probabilities that *Aedes* mosquitoes confer dengue infections to humans.

Heterogeneous Biting

Heterogeneous biting arises naturally from factors that contribute to aggregated feeding at multiple spatial scales; *e.g.*, in the neighborhood of aquatic habitats, at blood-feeding habitats, and on individuals. Among individual hosts, heterogeneous biting is characterized by γ, as already described. Among neighborhoods and blood-feeding habitats, heterogeneous biting is driven by spatial variation in the productivity of nearby aquatic habitats, the distribution of aquatic habitats and blood-feeding habitats relative to one another, and mosquito movement behavior. These factors can be combined into a vector of length $|f|$ that describes the expected number of bites per feeding cycle at each blood-feeding habitat:

$$M=\sum_{i=0}^{\infty}\bar{\Lambda}F(LF)^i, \quad (6)$$

where $\bar{\Lambda}$ is the average number of new adult females arising from each aquatic habitat per feeding cycle. Heterogeneous biting on the scale of individual hosts depends not only on spatial variation in biting intensity, but also on how individual hosts allocate their time at those locations and on the relative biting attractiveness of individuals at a location. Equipped with a matrix U that encapsulate these factors, we can define an $|f|$-by-$|h|$ matrix,

$$B=\text{diag}(M)U, \quad (7)$$

that gives the expected number of bites per feeding cycle on host i at blood-feeding habitat j. The notation diag(M) denotes a matrix with entries of the vector M along its diagonal and 0 elsewhere.

Pathogen Dispersion by Mosquitoes

Infected mosquitoes disperse pathogens across space as they make alternating movements between blood-feeding and aquatic habitats. Some mosquitoes might alternate repeatedly between a single blood-feeding habitat and a single aquatic habitat, whereas others might wander far from their natal aquatic habitat over the course of multiple feeding cycles. The movement paths that mosquitoes ultimately realize depend on their movement behavior, on their longevity, and on the spatial arrangement of blood-feeding and aquatic habitats. The distribution of paths along which pathogens are vectored by mosquitoes can be summarized with the $|f|$-by-$|f|$ matrix

$$Q=\sum_{i=\tau}^{\infty}(LF)^i, \quad (8)$$

which takes into account mortality and movement of mosquitoes between different habitat types after the τ feeding cycles required for pathogen incubation in the mosquito. Each row of Q thus gives the expected number of potentially infectious bites at each blood-

feeding habitat that originated from a single mosquito infected at a given blood-feeding habitat.

Pathogen Dispersion by Hosts

The dispersion of a pathogen from mosquitoes at one location to mosquitoes at another location by a mobile vertebrate host requires two bites on a single host: one at each location. One way to quantify this host-mediated element of pathogen dispersion is with the probability distribution of where secondary bites occur on an individual that received a primary bite at a given location, or

$$\Pr\{2^\circ \text{ bite at } j|1^\circ \text{bite at } i\}, \tag{9}$$

where i and j are elements of the set of blood-feeding habitats $\{f\}$ and Pr denotes the probability of a specified event. Successive bites must occur on a single host, however, so this probability must be further conditioned on each individual host $k \in \{h\}$, yielding

$$\Pr\{2^o \text{ bite at } j\ |1^o \text{ bite at } i\} = \sum_{k\in\{h\}} \Pr\{2^o \text{ bite at } j\ |2^o\text{bite on } k\}\Pr\{1^\circ\text{bite on } k|1^\circ\text{bite at } i\}. \tag{10}$$

The first probability on the right-hand side of eq. (10) is given by elements

$$W_{k,j} = \frac{B_{j,k}}{\sum_j B_{j,k}} \tag{11}$$

of an $|h|$-by-$|f|$ matrix W, while the second is given by $U_{i,k}$. The probability in eq. (20) for each i,j pair of blood-feeding habitats is then given by the i,j entry of the $|f|$-by-$|f|$ matrix

$$P = UW. \tag{12}$$

Each row of P then contains the expected distribution of where secondary bites occur on a host that received a primary bite at a given location.

Pathogen Amplification

Given the species-specific dispersion networks above, it is now possible to derive networks that describe pathogen dispersion through the entirety of the transmission cycle. Defining this cycle to begin and end in hosts, we obtain the expected number of secondary bites on each host arising from primary bites on a host k over the course of a single feeding cycle. Host k receives an average of $B_{i,k}$ bites per feeding cycle at blood-feeding habitat i. Each of the mosquitoes that bite at i then go on to make $Q_{i,\{f\}}$ bites at all the other blood-feeding habitats, which get distributed on hosts according to U. These steps combine mathematically to give an $|h|$-by-$|h|$ matrix,

$$V = B^T QU, \tag{13}$$

where ^{T} denotes transpose. In its entirety, the matrix V provides a description of the flow of mosquito biting from each host to every other host. Summing over all possible recipient hosts l for each primary host k yields

$$v_k = \sum_{l\in\{h\}} V_{k,l}, \tag{14}$$

which is the expected number of secondary bites on all hosts arising from primary bites on host k over the course of a single feeding cycle. The average of v_k over all $k \in \{h\}$ is the per-feeding-cycle analogue of the classical vectorial capacity metric, which measures per-host, daily pathogen amplification by mosquitoes, from Ross-Macdonald theory [71].

Host Infection

To describe how potentially infectious bites translate into new host infections, we must address at least three additional issues. First, the efficiency of pathogen transmission during a blood meal is not perfect. Mathematically, $V_{k,l}$ must be discounted by bc. Second, secondary bites on hosts arise repeatedly over $\bar{\rho}$ feeding cycles, on average, during which the primary host remains infectious. Third, consider that any number of infectious bites on a susceptible host l will have the same result: one and only one new host infection. We therefore introduce

$$R_{k,l} = 1 - e^{-bcV_{k,l}\bar{\rho}}, \tag{15}$$

which is the probability that host l receives one or more secondary infectious bites arising from host k [68]. The expected number of secondary infections on each host is also equal to $R_{k,l}$ and is thus subject to the reasonable bound of a maximum of one new infection per host. Summing over all possible secondary hosts, we see that the expected number of secondary infections on all hosts in a susceptible population arising from a single infection in host k is

$$r_k = \sum_{l\in\{h\}} R_{k,l}. \tag{16}$$

Each r_k is an individual-specific equivalent of the literal definition of the basic reproductive number R_0 from Ross-Macdonald theory; *i.e.*, the expected number of secondary host infections arising from a single infected host in an otherwise susceptible population [86]. As noted by [21], the dominant eigenvalue of R is equivalent to the definition of R_0 as a threshold for pathogen invasion and persistence.

Whereas R defines expected secondary infections from one host to another, we can also define a spatial analogue of R, which we call S, whose i,j entry specifies the expected number of secondary host infections at feeding habitat j that derive from a primary host infection incurred at feeding habitat i. This matrix is defined by the equation

$$S = URW, \tag{17}$$

which combines R with all the possible hosts that could incur an infection at i (U) and all the possible locations that secondarily infected hosts could incur their infections (W).

Supporting Information

Text S1 Additional details of the model and analysis.
(PDF)

Acknowledgments

We thank RC Reiner, TJ Hladish, and JRC Pulliam for feedback on the manuscript. Thanks to J Stoller for producing Fig. 1.

Author Contributions

Conceived and designed the experiments: TAP ALM DLS. Performed the experiments: TAP DLS. Analyzed the data: TAP DLS. Contributed reagents/materials/analysis tools: TAP TWS ALM DLS. Wrote the paper: TAP DLS.

References

1. MalERA (2011) A research agenda for malaria eradication: modeling. PLOS Medicine 8: e1000403.
2. ZMCP (2009) Malaria Elimination in Zanzibar: A Feasibility Assessment. Zanzibar Malaria Control Programme, 1–81 pp.
3. Tatem A, Smith D, Gething P, Kabaria C, Snow R, et al. (2010) Ranking of elimination feasibility between malaria-endemic countries. Lancet 376: 1579–1591.
4. Feachem R, Targett G (2009) Shrinking the Malaria Map: A Prospectus on Malaria Elimination. The Global Health Group, UCSF.
5. Smith T, Ross A, Maire N, Chitnis N, Studer A, et al. (2012) Ensemble modeling of the likely public health impact of a preerythrocytic malaria vaccine. PLOS Medicine 9: e1001157.
6. Cairns M, Roca-Feltrer A, Garske T, Wilson A, Diallo D, et al. (2012) Estimating the potential public health impact of seasonal malaria chemoprevention in African children. Nature Communications 3: 881.
7. Okell L, Griffn J, Kleinschmidt I, Hollingsworth T, Churcher T, et al. (2011) The potential contribution of mass treatment to the control of *Plasmodium falciparum* malaria. PLOS One 6: e20179.
8. Griffn J, Hollingsworth T, Okell L, Churcher T, White M, et al. (2010) Reducing *Plasmodium falciparum* malaria transmission in Africa: A model-based evaluation of intervention strategies. PLOS Medicine 7: e1000324.
9. Smith D, Hay S, Noor A, Snow R (2009) Predicting changing malaria risk after expanded insecticide-treated net coverage in Africa. Trends in Parasitology 25: 511–516.
10. Boni M, Smith D, Laxminarayan R (2008) Benefits of using multiple first-line therapies against malaria. Proceedings of the National Academy of Sciences 105: 14216–14221.
11. Smith D, Klein E, McKenzie F, Laxminarayan R (2010) Prospective strategies to delay the evolution of antimalarial drug resistance: weighing the uncertainty. Malaria Journal 9: 217.
12. Killeen G, Moore S (2012) Target product profiles for protecting against outdoor malaria transmission. Malaria Journal 11: 17.
13. Okumo F, Govella N, Moore S, Chitnis N, Killeen G (2010) Potential benefits, limitations and target product-profiles of odor-baited mosquito traps for malaria control in Africa. PLOS One 5: e11573.
14. Penny M, Maire N, Studer A, Schapira A, Smith T (2008) What should vaccine developers ask? Simulation of the effectiveness of malaria vaccines. PLOS One 3: e3193.
15. Ross R (1908) Report on the prevention of malaria in Mauritius. New York: E.P. Dutton & Company.
16. Ross R (1911) Some quantitative studies in epidemiology. Nature 87: 466–467.
17. Reiner R, Perkins T, Barker C, Niu T, Chaves L, et al. (2013) A systematic review of mathematical models of mosquito-borne pathogen transmission: 1970–2010. Journal of the Royal Society Interface 10: 20120921.
18. Hethcote H, Yorke J (1984) Gonorrhea Transmission Dynamics and Control. Springer-Verlag.
19. Keeling MJ, Eames KTD (2005) Networks and epidemic models. Journal of the Royal Society Interface 2: 295–307.
20. Stack J, Bansal S, Kumar V, Grenfell B (2012) Inferring population-level contact heterogeneity from common epidemic data. Journal of the Royal Society Interface 10:10120578.
21. Dye C, Hasibeder G (1986) Population dynamics of mosquito-borne disease: effects of flies which bite some people more frequently than others. Transactions of the Royal Society of Tropical Medicine and Hygiene 80: 69–77.
22. Woolhouse MEJ (1997) Heterogeneities in the transmission of infectious agents: implications for the design of control programs. Proceedings of the National Academy of Sciences 94: 338–342.
23. Lloyd-Smith JO, Schreiber SJ, Kopp PE, Getz WM (2005) Superspreading and the effect of individual variation on disease emergence. Nature 438: 355–359.
24. Smith D, Dushoff J, Snow R, Hay S (2005) The entomological inoculation rate and *Plasmodium falciparum* infection in African children. Nature 438: 492–495.
25. Smith DL, Drakeley CJ, Chiyaka C, Hay SI (2010) A quantitative analysis of transmission efficiency versus intensity for malaria. Nature Communications 1: 108.
26. Bousema T, Griffn J, Sauerwein R, Smith D, Churcher T, et al. (2012) Hitting hotspots: Spatial targeting of malaria for control and elimination. PLOS Medicine 9: e1001165.
27. Michael E, Ramaiah K, Hoti S, Barker G, Paul M, et al. (2001) Quantifying mosquito biting patterns on humans by DNA fingerprinting of bloodmeals. American Journal of Tropical Medicine and Hygiene 65: 722–728.
28. de Benedictis J, Chow-Shaffer E, Costero A, Clark GG, Edman JD, et al. (2003) Identification of the people from whom engorged *Aedes aegypti* took blood meals in Florida, Puerto Rico, using polymerase chain reaction-based DNA profiling. American Journal of Tropical Medicine and Hygiene 68: 437–446.
29. Scott TW, Githeko AK, Fleisher A, Harrington LC, Yan G (2006) DNA profiling of human blood in anophelines from lowland and highland sites in western Kenya. American Journal of Tropical Medicine and Hygiene 75: 231–237.
30. Port G, Boreham P, Bryan J (1980) The relationship of host size to feeding by mosquitoes of the *Anopheles gambiae* Giles complex (Diptera, Culicidae). Bulletin of Entomological Research 70: 133–144.
31. Carnevale P, Frezil J, Bosseno M, Le Pont F, Lancien J (1978) The aggressiveness of *Anopheles gambiae* A in relation to the age and sex of the human subjects. Bulletin of the World Health Organization 56: 147–154.
32. Walker E, Edman J (370–372) The inuence of host defensive behavior on mosquito (Diptera: Culicidae) biting persistence. Journal of Medical Entomology 22: 370–372.
33. Day J, Edman J (1984) Mosquito engorgement on normally defensive hosts depends on host activity patterns. Journal of Medical Entomology 21: 732–740.
34. Lindsay S, Ansell J, Selman C, Cox V, Hamilton K, et al. (2000) Effect of pregnancy on exposure to malaria mosquitoes. Lancet 355: 1972.
35. Ansell J, Hamilton K, Pinder M,Walraven G, Lindsay S (2002) Short-range attractiveness of pregnant women to *Anopheles gambiae* mosquitoes. Transactions of the Royal Society of Tropical Medicine and Hygiene 96: 113–116.
36. Shirai Y, Funada H, Seki T, Morohashi M, Kamimura K (2004) Landing preference of *Aedes albopictus* (Diptera: Culicidae) on human skin among ABO blood groups, secretors or nonsecretors, and ABH antigens. Journal of Medical Entomology 41: 796–799.
37. Shirai O, Tsuda T, Kitagawa S, Naitoh K, Seki T, et al. (2002) Alcohol ingestion stimulates mosquito attraction. Journal of the American Mosquito Control Association 18: 91–96.
38. Lefevre T, Gouagna L, Dabire K, Elguero E, Fontenille D, et al. (2010) Beer consumption increases human attractiveness to malaria mosquitoes. PLOS One 5: e9546.
39. Murphy M, Dunton R, Perich M, Rowley W (2001) Attraction of Anopheles (Diptera: Culicidae) to volatile chemicals in western Kenya. Journal of Medical Entomology 38: 242–244.
40. Takken W, Knols B (1999) Odor-mediated behavior of Afrotropical malaria mosquitoes. Annual Reviews of Entomology 44: 131–157.
41. Verhulst N, Qiu Y, Beijleveld H, Maliepaard C, Knights D, et al. (2011) Composition of human skin microbiota affects attractiveness to malaria mosquitoes. PLOS One 6: e28991.
42. Smallegange RC, Verhulst NO, Takken W (2011) Sweaty skin: an invitation to bite? Trends in Parasitology 27: 143–148.
43. Verhulst N, Andriessen R, Groenhagen U, Bukovinszkine Kiss G, Schulz S, et al. (2010) Differential attraction of malaria mosquitoes to volatile blends produced by human skin bacteria. PLOS One 5: e15829.
44. Lindsay S, Snow R (1988) The trouble with eaves; house entry by vectors of malaria. Transactions of the Royal Society of Tropical Medicine and Hygiene 82: 645–646.
45. Kirby M, Green C, Milligan P, Sismanidis C, Jasseh M, et al. (2008) Risk factors for house-entry by malaria vectors in a rural town and satellite villages in The Gambia. Malaria Journal 7.
46. Midega JT, Smith DL, Olotu A, Mwangangi JM, Nzovu JG, et al. (2012) Wind direction and proximity to larval sites determines malaria risk in Kilifi District in Kenya. Nature Communications 3: 674.
47. Carter R, Mendis KN, Roberts D (2000) Spatial targeting of interventions against malaria. Bulletin of the World Health Organization 78: 1401–1411.
48. Carter R (2002) Spatial simulation of malaria transmission and its control by malaria transmission blocking vaccination. International Journal for Parasitology 32: 1617–24.
49. Stoddard ST, Morrison AC, Vazquez-Prokopec GM, Paz Soldan V, Kochel TJ, et al. (2009) The role of human movement in the transmission of vector-borne pathogens. PLOS Neglected Tropical Diseases 3: e481.
50. Stoddard ST, Forshey BM, Morrison AC, Paz-Soldan VA, Vazquez-Prokopec GM, et al. (2013) House-to-house human movement drives dengue virus transmission. Proceedings of the National Academy of Sciences 110: 994–999.
51. Erhart A, Ngo DT, Phan VK, Ta TT, Van Overmeir C, et al. (2005) Epidemiology of forest malaria in central Vietnam: a large scale cross-sectional survey. Malaria Journal 4: 58.
52. Matthys B, Vounatsou P, Raso G, Tschannen A, Becket E, et al. (2006) Urban farming and malaria risk factors in a mediumsized town in Cote d'Ivoire. American Journal of Tropical Medicine and Hygiene 75: 1223–1231.
53. Dysoley L, Kaneko A, Eto H, Mita T, Socheat D, et al. (2008) Changing patterns of forest malaria among the mobile adult male population in Chumkiri District, Cambodia. Acta Tropica 106: 207–212.
54. Hairston N, de Meillon B (1968) On the ineffciency of transmission of *Wuchereria bancrofti* from mosquito to human host. Bulletin of the World Health Organization 38: 935–941.
55. Dietz K (1988) Mathematical models for transmission and control of malaria. In: Wernsdorfer, W and McGregor, I, editor, Principles and Practice of Malaria, Churchill Livingstone. pp. 1091–1133.

56. Koella J (1991) On the use of mathematical models of malaria transmission. Acta Tropica 49: 1–25.
57. Lloyd AL, Zhang J, Root aM (2007) Stochasticity and heterogeneity in host-vector models. Journal of the Royal Society Interface 4: 851–63.
58. Hasibeder G, Dye C (1988) Population dynamics of mosquitoborne disease: persistence in a completely heterogeneous environment. Theoretical Population Biology 33: 31–53.
59. Focks D, Daniels E, Haile D, Keesling J (1995) A simulation model of the epidemiology or urban dengue fever: literature analysis, model development, preliminary validation, and samples of simulation results. American Journal of Tropical Medicine and Hygiene 53: 489–506.
60. Magori K, Legros M, Puente M, Focks D, Scott T, et al. (2009) Skeeter Buster: a stochastic, spatially explicit modeling tool for studying *Aedes aegypti* population replacement and population supression strategies. PLOS Neglected Tropical Diseases 3: e508.
61. Depinay J, Mbogo C, Killeen G, Knols B, Beier J, et al. (2004) A simulation model of African Anopheles ecology and population dynamics for the analysis of malaria transmission. Malaria Journal 3: 29.
62. Smith T, Maire N, Dietz K, Killeen GF, Vounatsou P, et al. (2006) Relationship between the entomological innoculation rate and the force of infection for *Plasmodium falciparum* malaria. American Journal of Tropical Medicine and Hygiene 75: 11–18.
63. Bomblies A, Duchemin JB, Eltahir EaB (2008) Hydrology of malaria: Model development and application to a Sahelian village. Water Resources Research 44: 1–26.
64. Eckhoff PA (2011) A malaria transmission-directed model of mosquito life cycle and ecology. Malaria Journal 10: 303.
65. Chao DL, Halstead SB, Halloran ME, Longini IM (2012) Controlling dengue with vaccines in Thailand. PLOS Neglected Tropical Diseases 6: e1876.
66. Hii J, Smith T, Vounatsou P, Alexander N, Mai A, et al. (2001) Area effects of bednet use in a malaria-endemic area in Papua New Guinea. Transactions of the Royal Society of Tropical Medicine and Hygiene 95: 7–13.
67. Klinkenberg E, Onwona-Agyeman K, McCall P, Wilson M, Bates I, et al. (2010) Cohort trial reveals community impact of insecticide-treated nets on malariometric indices in urban Ghana. Transactions of the Royal Society of Tropical Medicine and Hygiene 104: 496–503.
68. Smith DL, McKenzie FE, Snow RW, Hay SI (2007) Revisiting the basic reproductive number for malaria and its implications for malaria control. PLOS Biology 5: e42.
69. Garrett-Jones C (1964) The human blood index of malaria vectors in relation to epidemiological assessment. Bulletin of the World Health Organization 30: 241–261.
70. Cosner C, Beier J, Cantrell R, Impoinvil D, Kapitanski L, et al. (2009) The effects of human movement on the persistence of vector-borne diseases. Journal of Theoretical Biology 258: 550–560.
71. Smith DL, Dushoff J, McKenzie FE (2004) The risk of a mosquito-borne infection in a heterogeneous environment. PLOS Biology 2: e368.
72. Onori E, Grab B (1980) Indicators for the forecasting of malaria epidemics. Bulletin of the World Health Organization 58: 91–98.
73. Hill M (1973) Diversity and evenness: a unifying notation and its consequences. Ecology 54: 427–432.
74. Favier C, Degallier N, Rosa-Freitas MG, Boulanger JP, Costa Lima JR, et al. (2006) Early determination of the reproductive number for vector-borne diseases: the case of dengue in Brazil. Tropical Medicine and International Health 11: 332–340.
75. Dietz K (1980) Models for vector-borne parasitic diseases. Lecture Notes in Biomathematics 39: 264–277.
76. Welch D, Bansal S, Hunter DR (2011) Statistical inference to advance network models in epidemiology. Epidemics 3: 38–45.
77. Johansson MA, Hombach J, Cummings DAT (2011) Models of the impact of dengue vaccines: a review of current research and potential approaches. Vaccine 29: 5860–5868.
78. WHO-VMI Dengue Vaccine Modeling Group (2012) Assessing the potential of a candidate dengue vaccine with mathematical modeling. PLOS Neglected Tropical Diseases 6: e1450.
79. Bomblies A, Duchemin JB, Eltahir EAB (2009) A mechanistic approach for accurate simulation of village scale malaria transmission. Malaria Journal 8: 223.
80. Linard C, Gilbert M, Snow R, Noor A, Tatem A (2012) Population distribution, settlement patterns and accessibility across Africa in 2010. PLOS One 7: e31743.
81. Kamdem C, Fouet C, Etouna J, Etoa FX, Simard F, et al. (2012) Spatially explicit analyses of anopheline mosquitoes indoor resting density: implications for malaria control. PLOS One 7: e31843.
82. Cummins B, Cortez R, Foppa IM, Walbeck J, Hyman JM (2012) A spatial model of mosquito host-seeking behavior. PLOS Computational Biology 8: e1002500.
83. Edman J, Scott T, Costero A, Morrison A, Harrington L, et al. (1998) *Aedes aegypti* (Diptera: Culicidae) movement inuenced by availability of oviposition sites. Journal of Medical Entomology 35: 578–583.
84. Vazquez-Prokopec G, Paz-Soldan V, Stoddard S, Morrison A, Elder J, et al. (2013) Linking fine scale human mobility and social contacts to understand infectious disease dynamics within a resource-poor urban center. PLOS One 8: e58802.
85. Scott T, Takken W Feeding strategies of anthropophilic mosquitoes result in increased risk of pathogen transmission. Trends in Parasitology 28: 114–121.
86. Smith DL, McKenzie FE (2004) Statics and dynamics of malaria infection in Anopheles mosquitoes. Malaria Journal 3: 13.
